# A vegetative storage protein improves drought tolerance in maize

**DOI:** 10.1111/pbi.13720

**Published:** 2021-12-08

**Authors:** Hari K. R. Abbaraju, Rajeev Gupta, Laura M. Appenzeller, Lynne P. Fallis, Jan Hazebroek, Genhai Zhu, Timothy M. Bourett, Richard J. Howard, Ben Weers, Renee H. Lafitte, Salim M. Hakimi, Jeffery R. Schussler, Dale F. Loussaert, Jeffery E. Habben, Kanwarpal S. Dhugga

**Affiliations:** ^1^ Corteva Agriscience Johnston IA USA; ^2^ AVX Corporation Fountain Inn SC USA; ^3^ USDA‐ARS Fargo ND USA; ^4^ Corteva Agriscience Hayward CA USA; ^5^ Corteva Agriscience Wilmington DE USA; ^6^ Corteva Agriscience Woodland CA USA; ^7^ Bill & Melinda Gates Foundation Seattle WA USA; ^8^ Corteva Agriscience Marion IA USA; ^9^ International Maize and Wheat Improvement Center (CIMMYT) El Batan Mexico

**Keywords:** chloroplast targeting, drought tolerance, grain yield, maize, mesophyll, nitrogen, remobilization, transgenic expression, vegetative storage protein

## Abstract

Vegetative storage proteins (VSPs) are known to serve as nitrogen reserves in many dicot plants but remain undiscovered in grasses, most widely grown group of crops globally. We identified and characterized a VSP in maize and demonstrated that its overexpression improved drought tolerance. Nitrogen supplementation selectively induced a mesophyll lipoxygenase (ZmLOX6), which was targeted to chloroplasts by a novel N‐terminal transit peptide of 62 amino acids. When ectopically expressed under the control of various tissue‐specific promoters, it accumulated to a fivefold higher level upon expression in the mesophyll cells than the wild‐type plants. Constitutive expression or targeted expression specifically to the bundle sheath cells increased its accumulation by less than twofold. The overexpressed ZmLOX6 was remobilized from the leaves like other major proteins during grain development. Evaluated in the field over locations and years, transgenic hybrids overexpressing ZmLOX6 in the mesophyll cells significantly outyielded nontransgenic sibs under managed drought stress imposed at flowering. Additional storage of nitrogen as a VSP in maize leaves ameliorated the effect of drought on grain yield.

## Introduction

Environmental stresses, which are becoming more severe and unpredictable with changing climate, substantially suppress crop yields below their potential levels (Wulff and Dhugga, 2018). The gap between the actual and potential yield must be narrowed to meet the demand for food grains from a growing and increasingly urbanized world population.

Increased application of nitrogen fertilizers has contributed to a substantial improvement in global food production over the last half century. Annual global demand for nitrogen fertilizers, which constitute one of the most expensive farm inputs, currently stands at about 117 million metric tonnes, with a projected future increase of ~1.5% per year (FAO, 2019). A considerable proportion of applied nitrogen is lost to leaching and runoff, particularly during seasons of excessive precipitation (Russo *et al*., 2017). Nitrogen runoff pollutes freshwater streams, causing excessive algal growth, which in turn lead to the development of dead zones in river deltas. When the dead organic matter decays, it asphyxiates aquatic life. Furthermore, excess nitrate leached into underground drinking water is unhealthy for humans and livestock. Another relatively minor route for nitrogen loss is denitrification (Hickman *et al*., 2014). Nitrous oxides released through denitrification, however, constitute potent greenhouse gases. Improving nitrogen use efficiency of crop plants, which would involve its faster removal from the soil and efficient utilization by the plant, could help reduce fertilizer input for maximal productivity and increase profitability of farm operations (Dhugga and Waines, 1989).

When reduced form of nitrogen like ammonium or urea is applied as a source of fertilizer, it is oxidized to nitrate by microbes in typical, aerated soils (Crawford and Glass, 1998). Most of the nitrate taken up by the plant is delivered to the root surface by mass flow. Under drought stress, thus, nitrogen uptake is also attenuated. After a drought episode ends, a recovery period ensues when water becomes available. Excess, dispensable form of stored nitrogen in the plant cells could recycle to buffer plant growth during the recovery period, filling the lag before the resumption of normal nitrate uptake and assimilation.

Crop plants with C3 photosynthesis are known to accumulate several‐fold more nitrogen in their leaves than C4 plants (Sinclair and Horie, 1989). The C3 plant wheat, for example, accumulates the entire nitrogen harvested at maturity by flowering in well‐fertilized and irrigated soils (Dhugga and Waines, 1989). In contrast, two‐thirds of the grain nitrogen in C4 maize is absorbed after flowering (DeBruin *et al*., 2013).

In addition to Rubisco, the most abundant protein in C3 plants, many plant species also store nitrogen temporarily as vegetative storage proteins (VSPs) when either the sink becomes limiting or leaves are shed prior to winter dormancy (Staswick, 1994). Before leaf shedding in deciduous trees, proteins are broken down into amino acids which are then transported to phloem in the branches and trunk, where they are sequestered in the form of VSPs such as alkaline phosphatases, chitinases, lectins and lipoxygenases, which range in size from approximately 15 to 100 kDa (Staswick, 1994). In spring, these VSPs are remobilized to initiate early shoot growth. When the terminal sink for nitrogen storage is limiting in annual crops like soybean, for example, upon pod removal, VSPs take up the role of nitrogen storage reserves in the leaves (Bunker *et al*., 1995; Dewald and Mason, 1992; Staswick, 1994). The occurrence of VSPs in grasses, the most widely cultivated group of crop plants globally, however, has not thus far been established (Grando *et al*., 2005).

Maize produces more biomass per unit land area than C3 crop plants but at half the nitrogen concentration in its vegetative tissues (Dhugga, 2007; Sinclair and Horie, 1989). In principle, then, it should be feasible to store additional organic nitrogen in the vegetative tissues of C4 plants like maize. Ability to store nitrogen in the form of VSPs would provide a sink in the plant for comparatively rapid removal of nitrogen from the soil, reducing its loss through leaching and runoff. Furthermore, these readily remobilized VSPs would buffer the plant against transient abiotic stresses. Another advantage of sequestering excess nitrogen in an osmoneutral form during periods of nitrogen abundance is the mitigation of feedback inhibition of continued nitrogen uptake and reduction by soluble nitrogen compounds such as amino acids (Tegeder and Masclaux‐Daubresse, 2018).

We undertook this study to identify potential VSP‐like proteins in maize, drive their ectopic expression with strong promoters, establish whether they remobilized during grain filling, and determine the effect of stored protein on plant performance in the field under normal commercial growing conditions as well as managed drought stress. We identified several soluble proteins in maize leaves that were selectively induced in response to the amount and form of nitrogen in the growth medium and demonstrated that the newly identified VSP, annotated as a lipoxygenase, ZmLOX6, was depleted from the leaves during grain development like other major leaf proteins. We further established that ZmLOX6 could be ectopically overexpressed at a fivefold higher level in the mesophyll cells than the wild‐type plants. Most importantly, maize hybrids overexpressing the ZmLOX6 gene under the control of the mesophyll cell‐specific p*ZmPEPC* promoter significantly outyielded their nontransgenic sibs under drought stress imposed at flowering in multi‐location, multi‐year field trials.

## Results and discussion

### Identification of maize leaf proteins induced by nitrogen

**Figure 1:**
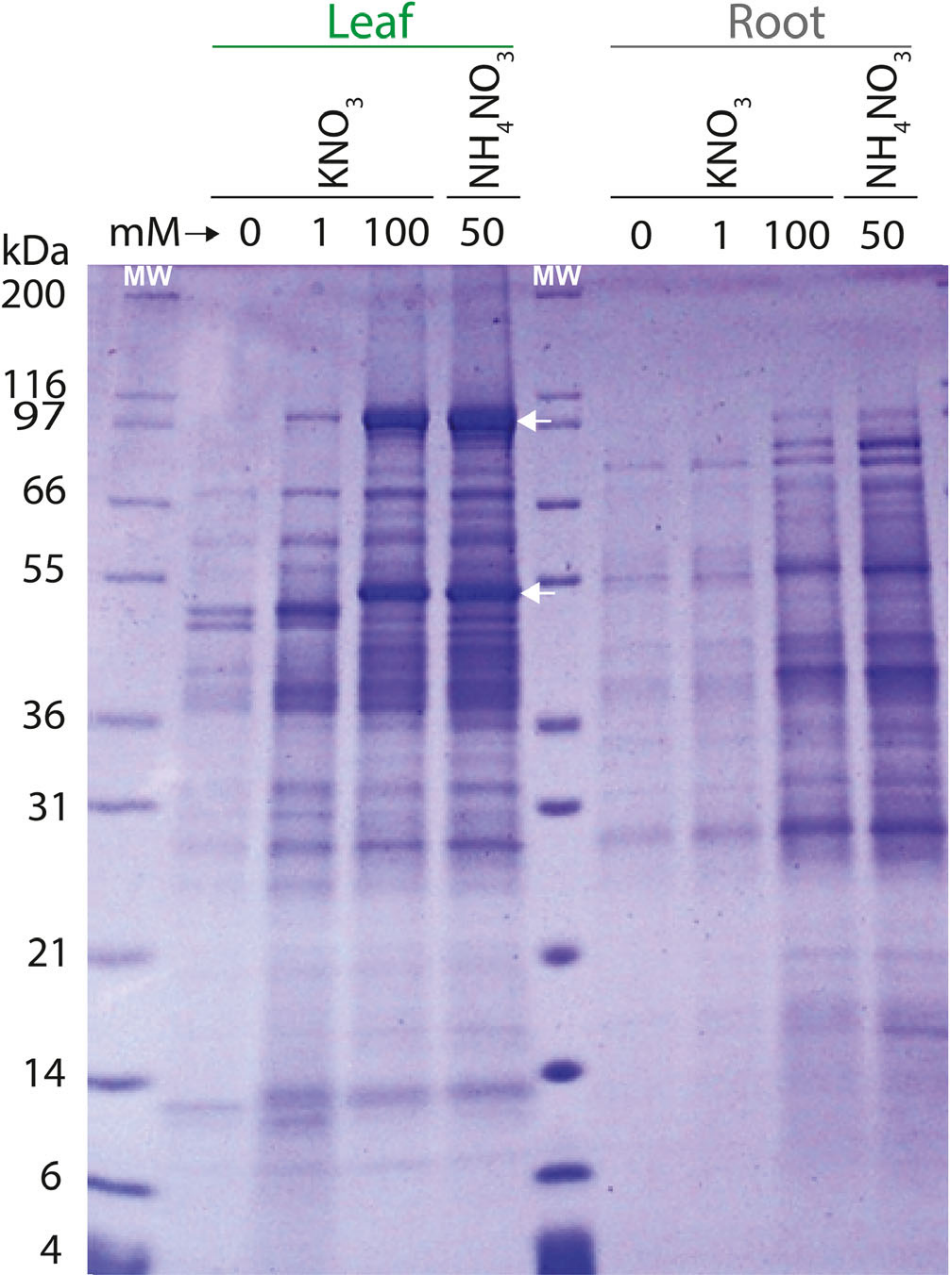
Induction of proteins in maize leaves (lanes 2–5) and roots (lanes 7–10) by potassium nitrate or ammonium nitrate in the growth medium. Maize seedlings were grown in vermiculite and irrigated with Hoagland solution containing no added nitrogen (lanes 2, 7), 1 mM potassium nitrate (lanes 3, 8), 100 mM potassium nitrate (lanes 4, 9) or 50 mM ammonium nitrate (lanes 5, 10). Lanes 1 and 6, molecular mass markers. Arrows point to major polypeptide bands induced in the leaves in response to nitrogen.

Two polypeptide bands of ~100 kDa and 50 kDa were selectively induced in the leaves of 11‐day‐old maize seedlings grown in nutrient media containing different concentrations of nitrogen supplied as potassium or ammonium nitrate (Figure 1 and Figure S1). Induction was more prominent with ammonium nitrate (50 mM) as compared to potassium nitrate (100 mM), even though the nitrogen concentration was the same between these two sources. Stronger induction could potentially be attributed to a higher mass percentage of nitrogen with ammonium as the counterion for nitrate (~35%) as compared to potassium (~14%), as well as enhanced bioavailability of the reduced form of nitrogen. Leaf blades rolled at approximately midday when 100 mM potassium nitrate was the source of nitrogen, suggesting osmotic stress from excess potassium. No leaf rolling was observed at equimolar nitrogen with ammonium nitrate as the fertilizer (Figure S1c,d).

Additional polypeptide bands were induced, particularly the one around 50 kDa (Figure 1). Induction was strongest in the fourth, fully expanded leaf from the base upward, although strong induction was also obvious in the fifth leaf, which was approaching full expansion (Figure S2). Nitrogen from the basal leaves had apparently already begun remobilizing, likely to the developing leaves near the apex, where the demand for reduced nitrogen was higher to support rapid cell expansion. Apparently, surplus nitrogen was stored in the cells in the form of proteins as a leaf approached full expansion and then recycled from the most mature to the rapidly expanding leaves. The leaves at positions 5 and 6 had sequentially lower amounts of the induced polypeptides, which suggested they were in the process of transitioning from sink to source leaves.

Peptides derived from a tryptic digest of the induced 100 kDa protein band matched four annotated proteins and a protein of unknown function. Aside from a lipoxygenase (ZmLOX6), a phosphoenol pyruvate carboxylase (ZmPEPC), a pyruvate orthophosphate dikinase (ZmPPDK) and an aconitate hydratase (aconitase) were identified (Figure S3). The 12 independent peptides, which together subsumed a total of 19 peptides, that matched ZmLOX6 ranged in length from 7 to 30 amino acids and together covered 17% of the polypeptide (Figure S4a,b). Intriguingly, no peptide from the N‐terminal stretch of 81 amino acids was detected (discussed in later sections).

**Figure 2:**
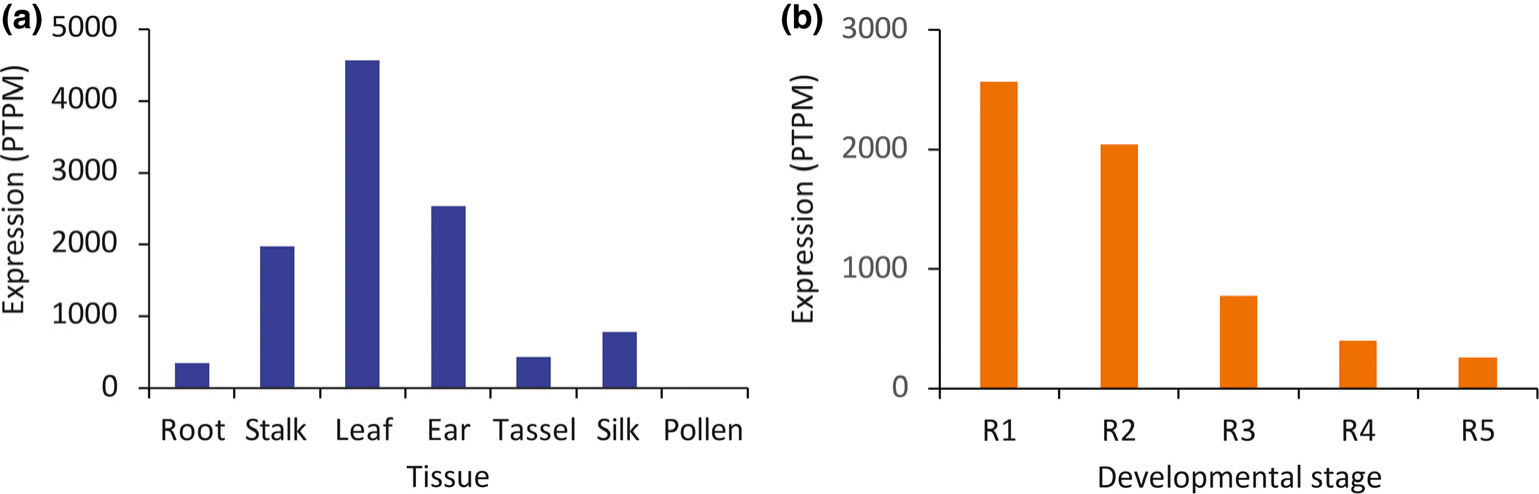
Expression of *ZmLox6* in maize tissues. The abundance of transcripts is expressed as protein‐coding transcripts per million (PTPM). The number of libraries for each of the tissues used to average the data was 28 for root, 2 for stalk, 53 for leaf, 19 for ear, 11 for tassel, 10 for silk and 2 for pollen (a). Each library was sampled on average four times. For the leaf on the ear‐bearing node, the data were averaged across 16 libraries each for the R1 to R3 stage and 15 libraries each for the R4 and R5 stages (b). A reduction in the expression of the *ZmLOX6* mRNA was commensurate with the log phase of grain filling, which starts at approximately the R2 stage, and follows the same trend as protein concentration in the ear leaf after flowering as shown in Figure 8.

ZmLOX6 is probably misannotated as a lipoxygenase based mainly on sequence similarity to the known enzymes of the same name. It stands out from the other lipoxygenases both by being genetically distant and also having lost the lipoxygenase activity (Gao *et al*., 2008). It has, however, retained the fatty acid hydroperoxide lyase activity, which had been previously described as a minor side activity of the main lipoxygenases. It is likely that this loss of primary lipoxygenase activity of ZmLOX6 is related to its evolution as a VSP. The *ZmLOX6* gene was primarily expressed in green tissues, most highly in the leaves, although it was also expressed at lower levels in other tissues, including roots (Figure 2a). It was not expressed in the pollen grains, however.

Whereas ZmPEPC and ZmPPDK are C4 enzymes and together constitute a little over 10% of the leaf protein (Sugiyama and Mizuno, 1984), aconitase is a tricarboxylic acid cycle enzyme that catalyses the isomerization of citrate to isocitrate. A dehydrogenase then converts isocitrate into α‐ketoglutarate, which constitutes the entry point for primary nitrogen assimilation into organic form via the glutamine synthetase–glutamate synthase cycle. Induction of aconitase by nitrogen suggested α‐ketoglutarate potentially constituted a limiting substrate in nitrogen assimilation.

Tryptic peptides from the induced polypeptide band at ~50 kDa matched a plastidial malate dehydrogenase (MDH), another enzyme in the C4 pathway (Silva *et al*., 2018) (Figure 1 and Figure S3). Induction of the three C4 enzymes, which together generate the C4 molecule malate from pyruvate, a C3 molecule, and carbon dioxide (bicarbonate), in response to nitrogen could be explained by the key reactions they catalyse in photosynthesis. It is also possible that these enzymes facilitate assimilation of excess reduced nitrogen through anaplerotic formation of aspartate and alanine from oxaloacetate and pyruvate, respectively, allowing for cycling of surplus ammonium in the cell directly into organic form. This cycle could operate to assimilate ammonium that exceeds the capacity of the glutamine synthetase–glutamate synthase cycle (Silva *et al*., 2017).

## Protein expression in E. coli, antibody production and ELISA development

Polyclonal antibodies were raised against the purified recombinant ZmLOX6, ZmPEPC and ZmPPDK proteins expressed in an *E. coli* expression system (Figures S5a,b). Anti‐ZmLOX6 antibody recognized a single polypeptide of ~100 kDa in the background of many proteins at a dilution of greater than 100,000‐fold on a Western blot (Figure 1, Figure S5b). Antibodies against ZmPEPC and ZmPPDK were similarly monospecific with titres exceeding 1:100,000 (data not shown).

Enzyme‐linked immunosorbent assays (ELISA) were developed and deployed to quantify each of the ZmLOX6, ZmPEPC and ZmPPDK proteins in total leaf protein extracts (Figure S6). Primary antibody dilutions of 10,000‐fold for anti‐ZmLOX6 and anti‐ZmPEPC, and 5,000‐fold dilution for the anti‐PPDK were subsequently used for the ELISA assays.

## Rationale for overexpression of ZmLOX6 to test its effect on plant performance

As to which of these induced polypeptides to focus on for further, in‐depth study, we relied on various lines of reasoning: relative association of each of the major leaf proteins to grain yield under normal irrigation and drought stress; expected pleiotropy upon ectopic expression of each of the proteins in the context of the respective reactions they catalysed in cellular metabolism; and possible limitation on the maximal amount of a protein that could be accumulated in a cell.

We quantified ZmLOX6, ZmPEPC and ZmPPDK proteins at flowering in the leaf at the ear‐bearing node using the respective ELISA assays in a set of 12 commercial hybrids grown under well‐watered conditions and managed drought in Woodland, CA. Under well‐watered conditions, ZmPEPC accounted for the most variation, 36%, in grain yield, followed by ZmLOX6 at 11%, with PPDK being inconsequential (Figures S7a,c,e). Under managed drought ZmLOX6 protein alone explained more than half of the variation, 58%, in grain yield (Figure S7b). In contrast, the proteins ZmPEPC and ZmPPDK both exhibited mild negative correlations with grain yield and explained relatively much less variation than ZmLOX6 (Figures S7d,f). The range in the respective concentrations of ZmPEPC and ZmPPDK in the leaf was narrower than that of ZmLOX6, which partly explained their relatively poor association with grain yield (Figure S7).

Path coefficient analysis, also referred to as multiple regression analysis of standardized variables, which takes into account the direct and indirect effects of independent variates ZmLOX6, ZmPEPC, and ZmPPDK on the dependent variable grain yield, was performed by the method of Wright (Wright, 1921) as simplified in Li (Li, 1975). It provided further support for the association of ZmPEPC with grain yield under well‐watered conditions (Figure S8a). Similarly, it highlighted the relatively strong association of ZmLOX6 with grain yield under drought stress where this protein alone accounted for 56% of the variation (Figure S8b). All three proteins together explained a total of 67% of the variation in grain yield.

ZmPPDK, ZmPEPC and ZmMDH, in that order, are the core enzymes of the C4 photosynthetic pathway. After ZmPPDK phosphorylates pyruvate, ZmPEPC carboxylates it to oxaloacetate, which ZmMDH then reduces to malate. Malate is transported into the bundle sheath cells where it is decarboxylated, and the resulting carbon dioxide then fixed by Rubisco. Pyruvate is shuttled back into the mesophyll cells to continue the C4 pathway. Aconitate hydratase is an enzyme of the tricarboxylic acid pathway. As all these enzymes catalyse key steps in metabolic pathways, we decided it was preferable not to alter their levels. Furthermore, at 4%–6% of the total leaf protein each, ZmPEPC and ZmPPDK proteins were already expressed at perhaps their cellular limits (detail in later sections), which contrasts with ZmLOX6 at less than 1% (Figure S9). Slightly higher concentrations of ZmPEPC and ZmPPDK had previously been reported in maize leaves (Sugiyama *et al*., 1984). The differences could be attributed to the antibodies and the methods used, for example, radial diffusion in the previous study as compared to ELISA in our case.

ZmLOX6, thus, appeared to be a logical candidate to store additional nitrogen in maize leaves. A previous study from soybean where a lipoxygenase was shown to perform the role of a VSP provided further support to explore this avenue (Tranbarger *et al*., 1991).

## Expression and induction of ZmLOX6 by nitrogen

ZmLOX6 was most abundant in the basal, one‐third of the developing leaf (Figure S9). The base of the maize leaf represents the zone of active cell division and expansion while the tip contains relatively mature cells, which are metabolically less active. For all subsequent studies involving leaf proteins, we thus sampled lamina on either side of the midrib from the basal third of the leaf.

**Figure 3:**
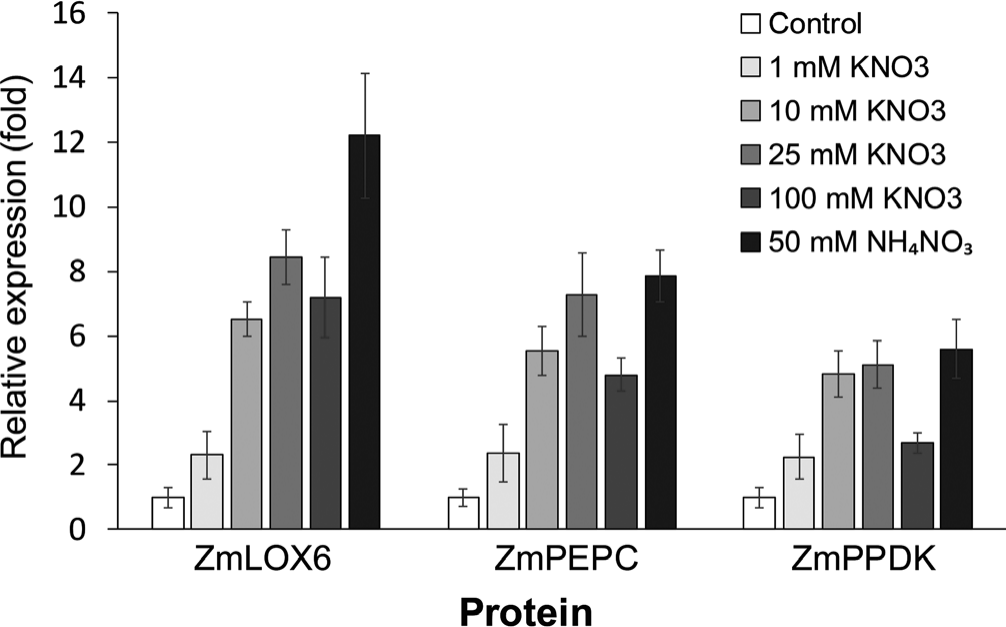
Quantification of protein induction by nitrogen in maize leaves. Protein extracts from the youngest, fully expanded leaves of 11‐day‐old plants grown in different forms and amounts of nitrogen were measured with ELISA. Concentrations of ZmLOX6, ZmPEPC and ZmPPDK in the total extractable leaf protein were 0.85%, 4.8% and 5.1%, respectively, in the plants supplemented with 10 mM KNO_3_. For fold expression, base level for each of the three proteins is set to 1.

In the source leaves of plants irrigated with a solution containing 5 mM nitrate, which is more than sufficient for optimal growth (Silva *et al*., 2017, 2018), ZmLOX6 constituted ~0.7% of the total leaf protein, whereas ZmPEPC and ZmPPDK each accounted for ~5% (Figure S7). To determine the extent of induction of proteins by various sources and amounts of nitrogen, the three proteins were quantified from leaf extracts of the A63 inbred maize line grown in the greenhouse. Although the other major leaf proteins, ZmPEPC and ZmPPDK, increased approximately 5‐ to 8‐fold in response to the highest nitrogen concentration, ZmLOX6 increased by up to 12‐fold (Figure 3). When grown in potassium nitrate, however, ZmPEPC and ZmPPDK accumulated at a lower level at 100 mM than at the 25 mM concentration, possibly because of osmotic stress from excess potassium. At equimolar nitrogen with ammonium nitrate as the source, protein induction was not suppressed. However, except for ZmLOX6, induction of which was notably enhanced, no further accumulation was observed for ZmPEPC and ZmPPDK than at 25 mM potassium nitrate. Readily available reduced form of nitrogen from ammonium nitrate could enhance the formation of amino acids, which in excess could be stored in the form of ZmLOX6 and other proteins. These results support our earlier suggestion that there may be an upper limit on the levels of ZmPEPC and ZmPPDK that could accumulate in the leaves. In contrast, ZmLOX6 accumulated further in response to reduced form of nitrogen, not only likely because it was expressed at a comparatively low level to begin with but also perhaps because it was a dispensable protein with no important biological function other than nitrogen storage.

## Cellular and intracellular localization of ZmLOX6 in maize leaves

ZmLOX6 accumulated solely in mesophyll cells as seen from its expression by Western blotting of protein extracts from dissected vascular bundles and mesophyll tissue, suggesting that the site of its accumulation was away from the site of carbon fixation, which is confined to the bundle sheath cells in C4 plants (Figure S10). Within the mesophyll cells, ZmLOX6 was localized to chloroplasts as judged from immunocytochemical localization with the anti‐ZmLOX6 antibody (Figure S11a). It was expressed at a low level in the roots as well and there, too, it localized to plastids (Figure S11b).

To understand its transport into the plastids, we searched but were unable to identify a transit signal peptide with any of the known *in silico* prediction methods. Similarly, ZmLOX6 was previously reported not to have a discernible signal peptide that could be identified by the prediction algorithms but was shown to be targeted to the chloroplasts by *in vitro* transport assays (Gao *et al*., 2008).

## Identification and validation of a novel chloroplast targeting signal peptide in ZmLOX6

Before subjecting to mass spectrometry, the 100 kDa polypeptide band was originally digested with trypsin, which cleaves proteins C‐terminal to the arginyl and lysyl residues. Absence of any peptide in the N‐terminal stretch of 81 amino acids in the tryptic digest suggested that the signal peptide was most likely present within this region (Figure S4). Trypsin cleaved ZmLOX6 at approximately one‐fifth (19 of 86) of the remaining target residues. If the nitrogen‐induced ZmLOX6 in the chloroplast were to remain unprocessed, at least one and likely two peptides should have been detected by mass spectroscopy in the region corresponding to the N‐terminal 81 amino acids as it contained 9 of the 95 arginyl and lysyl residues present in polypeptide. This suggested that at least a portion, if not all, of the first 81 amino acids acted as a targeting signal. A construct consisting of an in‐frame fusion of this portion of ZmLOX6 with a green fluorescent protein derived from *Aequorea coerulescens* (AcGFP) was introduced into young maize leaves by biolistic transformation for transient expression. Indeed, the fluorescence was targeted to the chloroplasts (data not shown).

To determine the precise cleavage site of the signal peptide, we immunopurified the ZmLOX6 protein from the leaves assuming it must already be processed (Figure S12). N‐terminal sequencing returned 14 amino acids from the purified polypeptide, which matched the amino acids 63–76 of the ZmLOX6 protein as predicted from the translation product of the cDNA, indicating that the signal peptide cleavage occurred C‐terminal to the 62^nd^ amino acid, an arginine (Figure S4).

**Figure 4:**
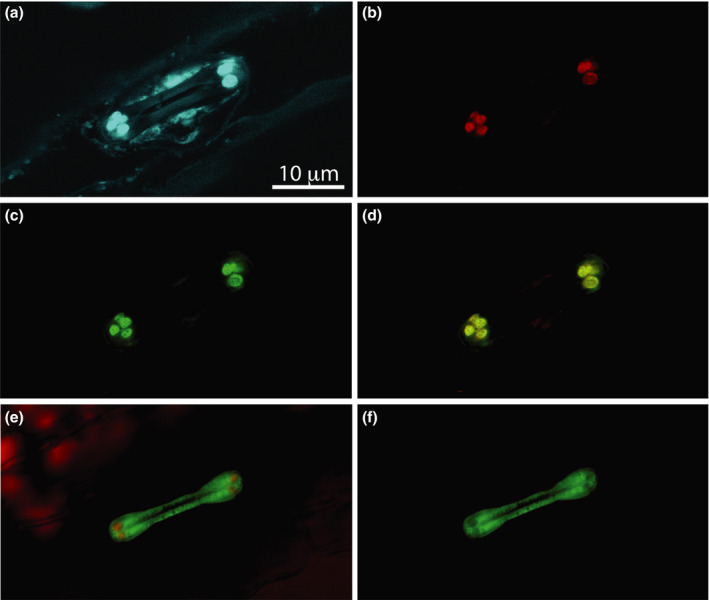
Validation of chloroplast targeting peptide for ZmLOX6. Precise cleavage site of the signal peptide was determined by N‐terminal sequencing of the immunopurified mature ZmLOX6 protein from maize leaves. DNA encoding 1–62 amino acids long target peptide was ligated to a green fluorescent protein (AcGFP) gene and transformed into maize seedling leaves for transient expression (a‐d). Guard cell pair of a single stomate is visible when autofluorescence is overlaid with chlorophyll fluorescence (a); (b) chlorophyll fluorescence alone; (c) AcGFP fluorescence alone; (d) chlorophyll fluorescence overlaid with AcGFP fluorescence; (e) chlorophyll and AcGFP fluorescence together with a 60 amino acids long signal peptide fused to AcGFP and (f) AcGFP fluorescence alone with the 60 amino acids long signal peptide. AcGFP was targeted to cytoplasm when the signal peptide length was truncated by 2 amino acids to 60 amino acids (e and f).

A construct encoding a fusion protein consisting of N‐terminal 60 or 62 amino acids of ZmLOX6 and AcGFP, the expression of which was driven by the p*Ubi‐intron* promoter, was then introduced into young maize leaves by biolistic transformation. Transient expression was best observed in the guard cells of stomata (Figures 4a–c). Unlike plastids in the pavement cells of the epidermis, which contained too little chlorophyll to image, the high chlorophyll content of the guard cells made it possible to readily image the chloroplasts. When the AcGFP fusion protein contained only the N‐terminal 60 amino acids, it was distributed throughout the cytoplasm (Figures 4e,f). Upon in‐frame fusion with the N‐terminal 62 amino acids, however, the AcGFP specifically localized to the chloroplasts, demonstrating that this stretch of amino acids constituted the signal peptide for transport of ZmLOX6 into the chloroplasts (Figures 4c,d). This signal peptide was unique as the only protein it matched in the publicly available databases was ZmLOX6.

## Effect of different promoters and a vacuolar targeting signal on expression of ZmLOX6

With the underlying hypothesis that surplus reduced form of nitrogen would be available during peak photosynthesis in the plants grown in well‐fertilized and irrigated soils, our objective was to express high levels of ZmLOX6 ectopically in the leaves to sequester excess cellular nitrogen in an osmoneutral form. Subsequently, the stored nitrogen could be remobilized during periods of deficiency, for example, after transient or prolonged drought stress.

**Figure 5:**
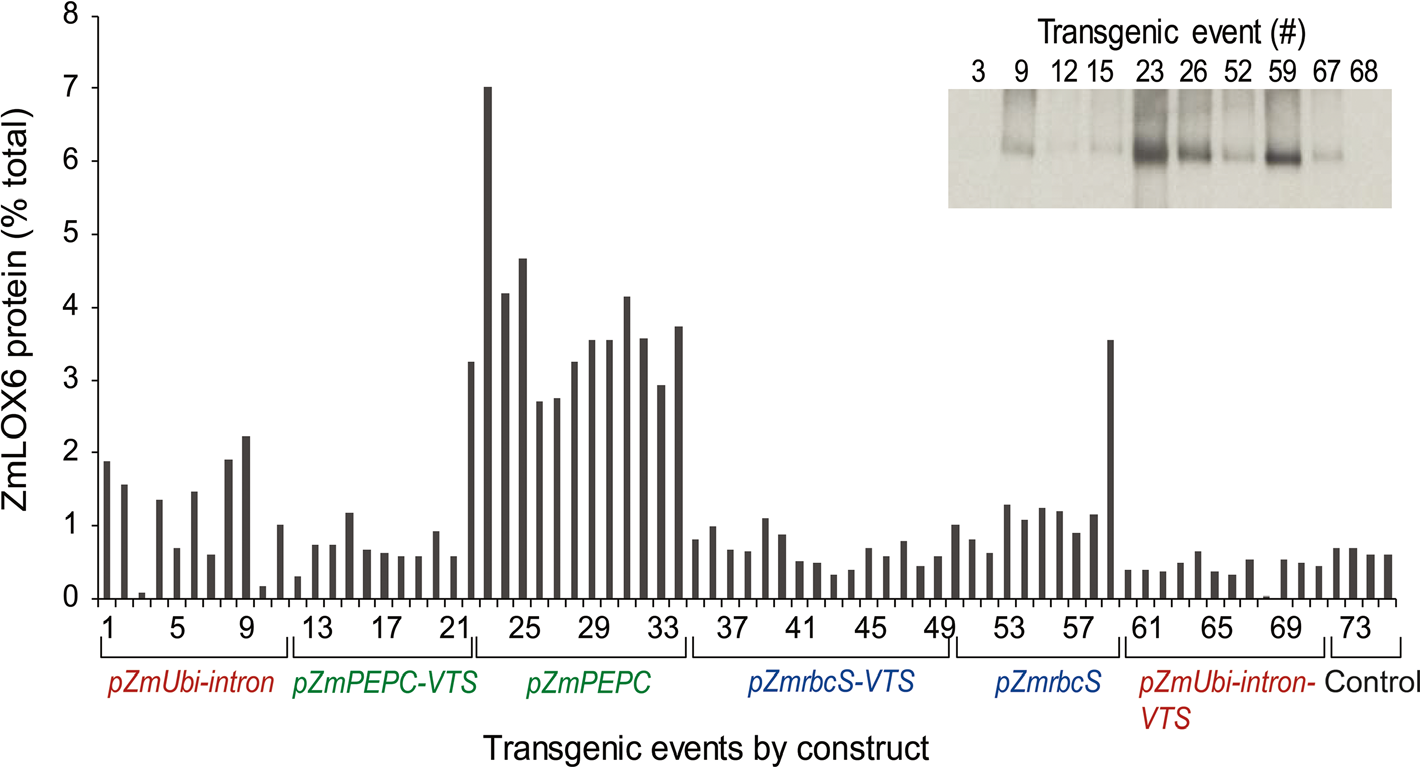
Quantification of leaf ZmLOX6 protein in transgenic and control maize plants by ELISA. *ZmLOX6* gene was transformed under the control of different promoters with or without a vacuolar‐targeting signal (VTS) from maize aleurain. Protein extracts from the youngest, most fully expanded leaf of 11‐day‐old plants grown in 5 mM potassium nitrate were subjected to ELISA with the anti‐ZmLOX6 antibody. A subset of representative events corresponding to the numbers on the abscissa with varying ELISA signal were probed with the anti‐ZmLOX6 antibody on a Western blot (inset). The constructs with each of the three promoters with or without the VTS are colour coded. Protein level displayed on the ordinate is expressed as percent of the total extractable leaf protein.

To determine which cell type allowed maximal accumulation, we expressed the complete open reading frame of the ZmLOX6 cDNA under the control of three different promoters: p*ZmUbi‐intron* for constitutive expression, maize phosphoenol pyruvate carboxylase (p*ZmPEPC*) for expression in mesophyll cells and maize Rubisco small subunit (p*ZmrbcS*) for expression in the bundle sheath cells after confirming their respective expression patterns using AcGFP fusions, which agreed with the previous reports (Christensen and Quail, 1996; Kausch *et al*., 2001; Schaffner and Sheen, 1991) (data not shown). A VSP‐like lipoxygenase in soybean was earlier reported to accumulate in the vacuole (Tranbarger *et al*., 1991). At the time of these experiments, we had not yet determined the cellular or intracellular localization of ZmLOX6. Thus, in addition to the constructs with the three promoters, we also fused a vacuolar targeting signal (VTS) from maize aleurain in a parallel set of constructs (Griffiths *et al*., 1997; Holwerda and Rogers, 1993). From a subset of single‐copy, transgene expressing events, protein isolated from the youngest fully expanded leaf at five‐leaf stage was assayed with ELISA (Figure 5). To corroborate the ELISA results, an immunoblot was generated from the leaf extracts of several transgenic events that accumulated varying levels of ZmLOX6 (Figure 5, inset). Semi‐quantitative Western blot results were in complete agreement with the ZmLOX6 concentrations measured with quantitative ELISA. Base‐level expression of ZmLOX6 as seen in events 72–75 was ~0.7% of the total leaf protein (Figure 5). The ZmLOX6 protein accumulated to the highest level, up to 10‐fold higher than the controls, in plants expressing the gene under the control of the p*ZmPEPC* promoter but without the *VTS* (Figure 5, events 22–34). Its expression under the control of p*ZmUbi‐intron* (events 1‐11) or p*ZmrbcS* (events 50‐59) promoters also led to higher levels of the protein accumulation but the increase on average was only twofold. A striking observation was that the fusion of the VTS at the N‐terminus of the predicted protein adversely affected the accumulation of the protein regardless of the promoter used (Figure 5, events 12–21, 35–49 and 60–71). Evidently, the VTS interfered with the chloroplast targeting signal of ZmLOX6.

Expression of a dicot VSP, soybean VSPβ, in maize under the control of the p*ZmUbi‐intron* promoter resulted in its accumulation to a level of 0.5% of the total leaf protein in the primary transgenic plants. In the following generation, the expressed protein accumulated only to a level of 0.03% even though mRNA was present in the cells (Grando *et al*., 2005). Translation efficiency of a nonmaize gene was apparently the cause for low‐level expression of the soybean VSP.

**Figure 6:**
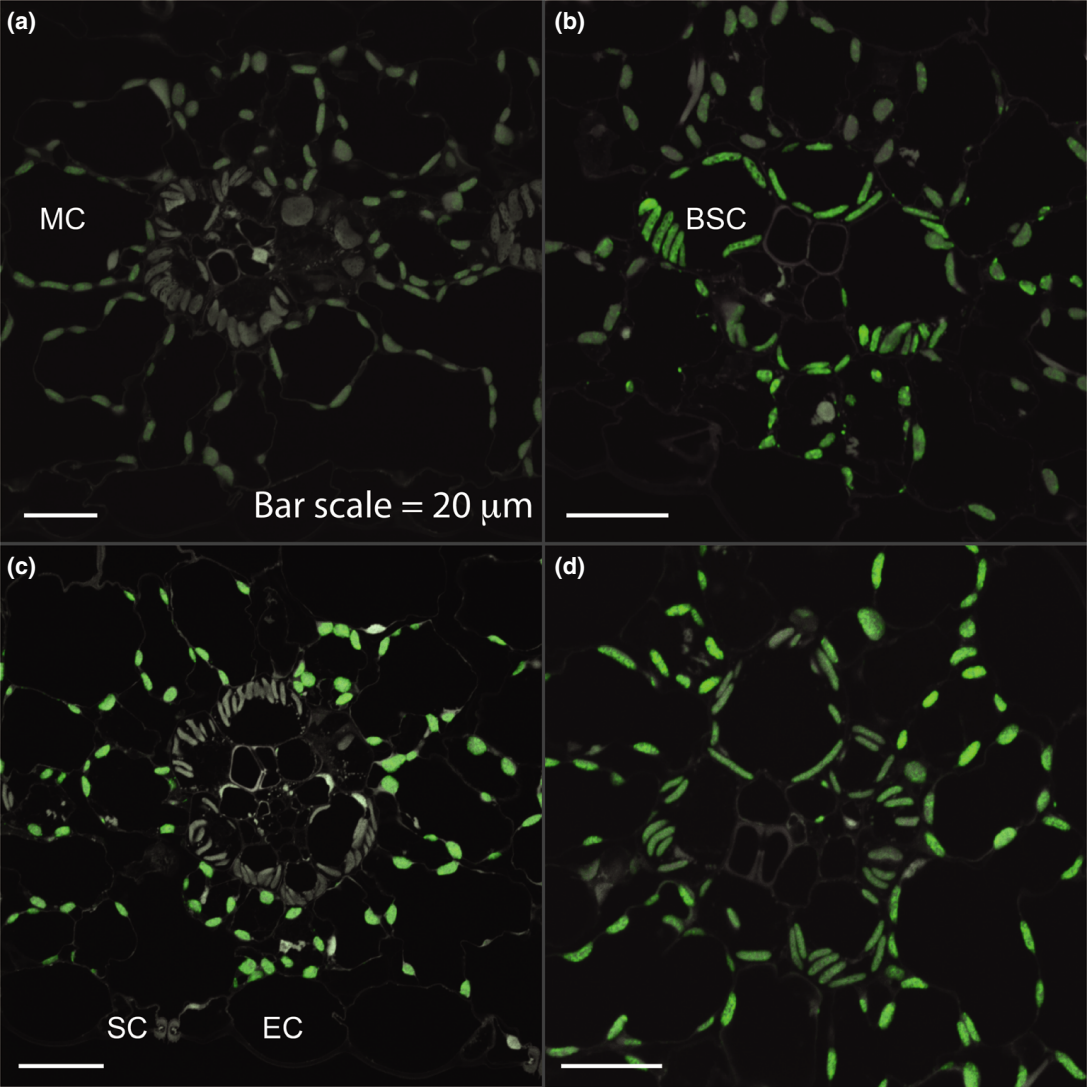
Immunocytochemical localization of ZmLOX6 protein in maize seedling leaves derived from: wild‐type plants (a); transgenic plants expressing *ZmLOX6* under the control of p*ZmrbcS* (b), p*ZmPEPC* (c) and p*ZmUbi‐intron* (d) promoters. The ZmLOX6 protein is expressed in mesophyll cells at low level in native cells, thus the background signal (a). Abbreviations: BSC, bundle sheath cell; EC, epidermal cell; MC, mesophyll cell; and SC, stomatal cells.

**Figure 7:**
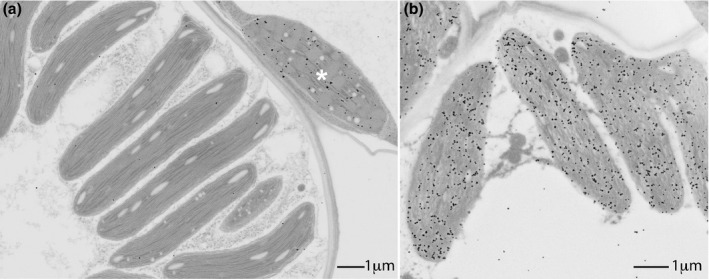
High‐resolution localization of the ZmLOX6 protein in maize seedling leaves from (a) a wild‐type plant and (b) a transgenic plant expressing the *ZmLOX6* gene under the control of the Rubisco small subunit (p*ZmrbcS*) promoter. In the wild‐type leaf, ZmLOX6 is localized to a single, visible mesophyll chloroplast (asterisk), in contrast to the unlabelled, appressed chloroplasts in an adjacent bundle sheath cell (a).

Cell specificity of each promoter was determined by immunolocalization of the ZmLOX6 protein in the transgenic events (Figures 6,7). As expected, the p*ZmrbcS* promoter was only expressed in the bundle sheath cells (Figures 6b,7b), p*ZmPEPC* only in the mesophyll (Figure 6c) and p*Ubi‐intron* in both the cell types (Figure 6d).

The p*ZmPEPC* promoter was not expressed in the stomatal guard cells, at least not to the extent that it could be detected with microscopy (Figures S13, 14). In contrast, the expression of the p*ZmrbcS* promoter could be detected in the guard cell chloroplasts (Figure S14). These expression patterns are in agreement with a previous study where only the p*ZmrbcS* promoter was reported to be expressed in the guard cells but not the p*ZmPEPC* promoter (Sattarzadeh *et al*., 2010). In the absence of micrographs from the stable transgenic plants generated with the p*Ubi‐intron* promoter, we do not know whether this promoter was expressed in the stomatal guard cells in stable transgenics. The observation that it was indeed active in these cells upon transient expression after biolistic transformation suggests, however, that it was most likely expressed (Figure 4).

**Figure 8:**
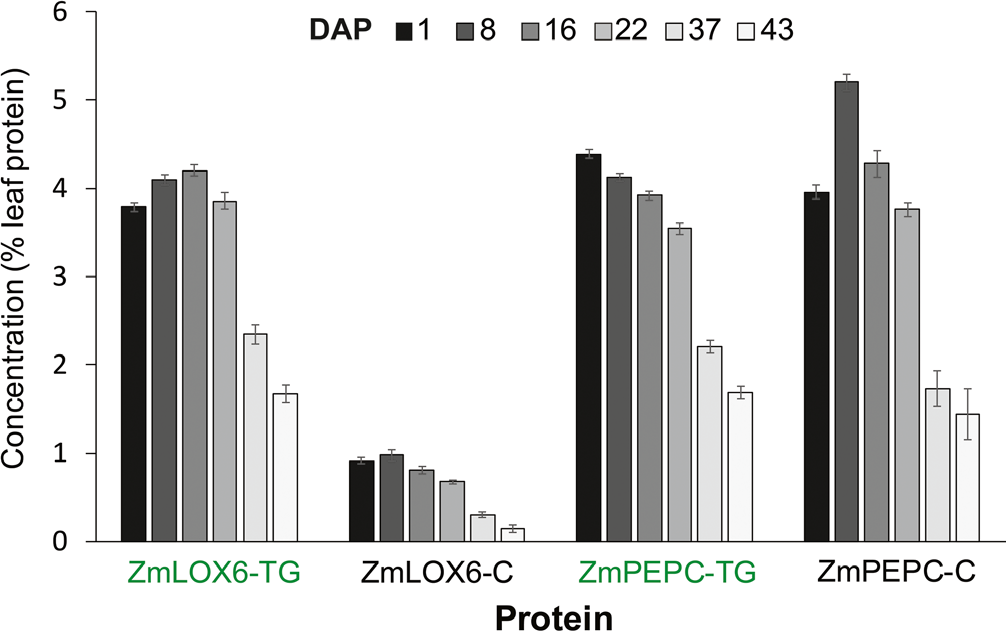
Remobilization of ZmLOX6 and ZmPEPC proteins after pollination from the plants overexpressing *ZmLox6* under the control of the p*ZmPEPC* promoter. Ear leaves of field‐grown 16 independent transgenic events overexpressing *ZmLOX6* under the control of the p*ZmPEPC* promoter and the parental nontransgenic line were repeatedly sampled at different days after pollination (DAP). The protein extract was subjected to ELISA for the ZmLOX6 and ZmPEPC proteins. The suffix TG after ZmLOX6 and ZmPEPC refers to *ZmLOX6*‐overexpressing transgenic plants (green font) and the suffix C refers to nontransgenic control plants (black font). The ELISA‐quantified proteins are expressed as a percentage of the total extractable leaf proteins at each stage.

ELISA indicated that ZmPEPC was one of the most abundant proteins in the leaves of field‐grown nontransgenic maize plants, constituting 4%–5% (w/w) of the total leaf protein (Figure 8 and S7). ZmLOX6 constituted less than 1% of the total leaf protein in nontransgenic plants (Figure 8). In transgenic events driven by the p*ZmPEPC* promoter, ZmLOX6 accumulated to the same level as the most abundant leaf proteins ZmPEPC and ZmPPDK (Figures 5 and 8).

**Figure 9:**
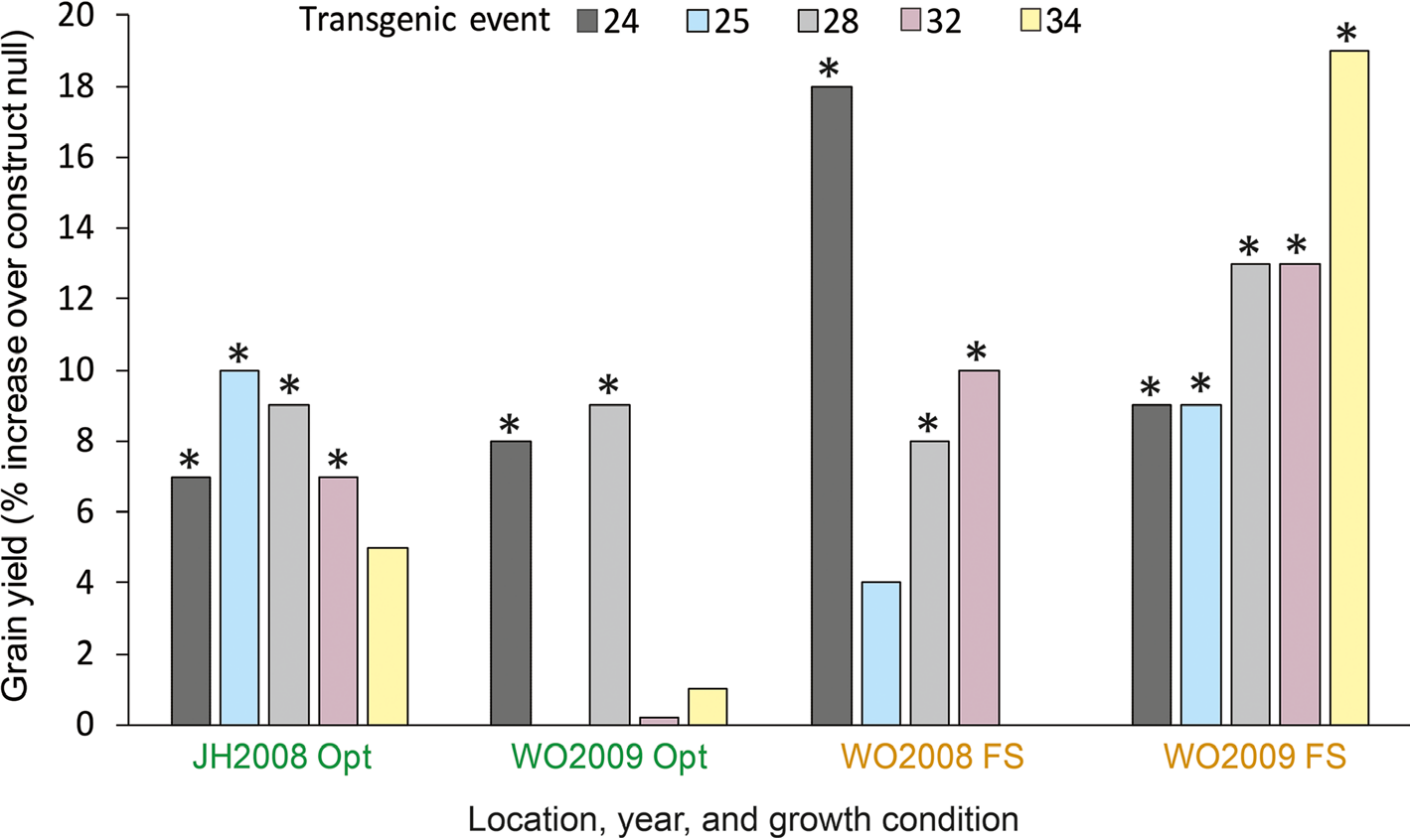
Field performance of transgenic hybrids overexpressing *ZmLOX6* under the control of the p*ZmPEPC* promoter as compared to their nontransgenic sibs across locations (JH, Johnston, IA; WO, Woodland, CA), years and water stress (Opt, optimal conditions; FS, flowering drought stress) with eight replications of four‐row plots under drought stress and four to six replications under optimal conditions. Events that yielded significantly more grain than the null comparator are labelled with asterisks. Event 34 was excluded from the year 2008 under water stress because of its failure in multiple replications. Null comparator grain yield in 2008 under optimal conditions was 12.7 t.ha^‐1^ in Johnston, IA, and 13.9 t.ha^‐1^ in Woodland, CA. Application of drought stress at flowering caused an approximately 25% reduction in grain yield. The labels for optimal and drought stress treatments are colour coded in green and brown fonts respectively.

Transgenic events 23 and 59, which were derived from the overexpression of the ZmLOX6 gene under the control of the p*ZmPEPC* and p*ZmrbcS* promoters, respectively, accumulated the highest amounts of the ZmLOX6 protein among the events within each group (Figure 5). Neither survived beyond the five‐leaf stage, suggesting an upper limit to the accumulation of an ectopically expressed protein in each of the cell types, which was approximately 5% for the mesophyll cells and 1.5% for the bundle sheath cells (Figure 5). Two events, 3 and 68, were apparently silenced as shown by the ELISA assays and Western analysis (Figure 5), likely because of co‐suppression, which is a common phenomenon in transgenic expression. Up to 10% of the transgenic events targeted for overexpression could silence the native gene as well (Dhugga K., unpublished data). The silenced seedlings looked normal, however, suggesting that ZmLOX6 was a dispensable protein.

## Remobilization of ZmLOX6 from leaves after flowering

As grain development progresses, leaf senescence sets in around R3–R4 stage (DeBruin *et al*., 2013). Senescence is the result of degradation of leaf proteins and remobilization of amino acids to the grain, an evolutionary mechanism to conserve nitrogen (DeBruin *et al*., 2013; Dhugga and Waines, 1989). To ascertain whether ZmLOX6 was remobilized like other leaf proteins, we performed a field experiment to measure its concentration in the leaf from the ear‐bearing node starting at flowering and ending at maturity on 16 field‐grown transgenic events overexpressing *ZmLOX6* under the control of the p*ZmPEPC* promoter and the nontransgenic parental line (Figure 8). As expected, the ZmLOX6 protein remobilized from the leaf just like another major leaf protein, ZmPEPC. Similarly, VSPs were shown to be remobilized preferentially during seed development in soybean (Staswick, 1989). The upper limit for nitrogen remobilization from the leaf in maize is a little over 60% regardless of the starting amount, which is further supported by these results (DeBruin *et al*., 2013).

## Hybrids overexpressing ZmLOX6 outperformed control hybrids under managed drought stress

We had advanced the hypothesis that readily available osmoneutral form of stored nitrogen would confer yield advantage on maize hybrids under drought stress as it could be recycled for the formation of enzyme proteins during the recovery phase after water became available. Single‐copy transgenic events expressing *ZmLOX6* driven by the p*ZmPEPC* promoter were crossed to a tester inbred line and the hybrids evaluated at two locations for 1 year and over 2 years in one location in well‐replicated trials with eight replications under drought stress and four to six under normal conditions (details in M&M). Water stress was managed at the Woodland (California) location in both years. The location in Johnston (Iowa), although rain fed, is optimal for grain yield as frequent precipitation provides sufficient moisture for normal crop growth. A bulked transgenic null, which consisted of nontransgenic seed derived from the same plants as for transgenic seed and thus had the same genetic background, served as control.

**Figure 10:**
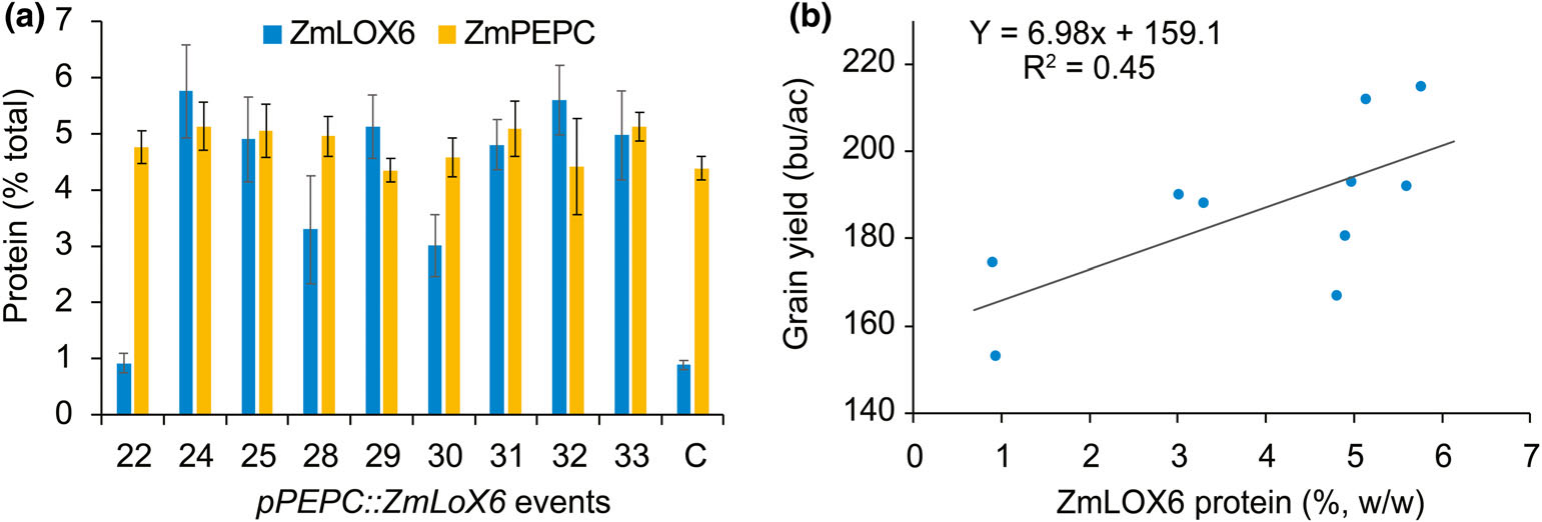
Relationship of ZmLOX6 protein in *pZmPEPC‐*driven *ZmLOX6* transgenic events with grain yield. ZmLOX6 protein is presented as a percentage of the total leaf protein in the ear leaf 2 weeks before flowering in Woodland, CA (2008) (a), and its relationship to grain yield under drought stress at flowering (b). The ear leaf was at the 8^th^ position of the aboveground nodes. Leaf discs were collected as described in Experimental Procedures and frozen immediately in dry ice. The leaf extracts were subjected to ELISA using the anti‐LOX6 antibody. One of the ten transgenic events, #34, failed in 2008 so was omitted from the graph. Another event, #1, was apparently completely silenced as it had the same level of ZmLOX6 as the construct null. Six of the transgenic events had similar concentration of the ZmLOX6 protein but two had significantly lower concentration, although they still accumulated three times as much protein as the construct null. Grain yield under drought stress was positively correlated with the ZmLOX6 protein concentration measured before the application of stress. ZmPEPC protein, which was measured on the same extracts as for the ZmLOX6 protein, is shown for reference. Its levels were unaffected by those of ZmLOX6.

Several transgenic events outperformed the construct null (Figure 9). Four of the ten events had superior grain yield under flowering stress in Woodland and three were significantly better than the construct null in each of the 2 years (Figure 9). At error set to an alpha level of 0.1, only one of the 10 transgenic events was expected to be a false positive within each testing environment. The chances of the same event being a false positive across 2 years in the same location would then be one out of a hundred and those of three independent events performing consistently across 2 years by chance essentially negligible, one out of a million. Three of the ten events outperformed the construct null in both years, giving us the confidence that ZmLOX6 overexpression buffered the plants against drought stress. Furthermore, overexpression of ZmLOX6 did not impose any yield penalty under normal growing conditions (Figure 9). Other plant characteristics such as time to silk and pollen shed, plant height and grain moisture were not affected (Table S1). Grain yield performance of the transgenic hybrids under drought stress was generally positively associated with the ZmLOX6 protein level in the ear leaves (Figure 10). The *ZmLOX6* gene in the hybrid derived from event 1 appeared to have been silenced, a phenomenon known to cause event‐to‐event variation in transgenic plants (Matzke and Matzke, 1998).

Down‐regulation of soybean VSPs had no adverse impact on plant performance (Staswick *et al*., 2001). Leaf nitrogen was maintained at normal levels, however, suggesting its storage in other forms (Staswick *et al*., 2001). As discussed earlier, under normal soil nitrogen, C3 plants store nearly all the nitrogen required for grain development by flowering, whereas C4 plants acquire two‐thirds of the required grain nitrogen after flowering (DeBruin *et al*., 2013; Dhugga and Waines, 1989). In addition, osmoneutral form of nitrogen in the maize hybrids overexpressing the ZmLOX6 protein apparently stabilized and improved their performance, particularly under water stress.

To address whether the additional ZmLOX6 protein affected plant performance simply by conserving more nitrogen or enhancing plant growth, we conducted a study under controlled conditions involving seedlings of the transgenic events. Analyses of 18‐day‐old seedlings of multiple single‐copy, semi‐hydroponically grown transgenic events showed that, whereas expression of the transgene in the bundle sheath cells increased tissue nitrogen concentration, it adversely impacted biomass (Table S2 and Figure S15). Expression in the mesophyll cells, in contrast, increased total biomass and total nitrogen but did not in general affect tissue nitrogen concentration. These results point to different metabolic fates of the stored osmoneutral form of nitrogen in the two cell types (Figure 5, Table S2 and Figure S15). It is possible that ectopic expression of ZmLOX6 in the bundle sheath chloroplasts, where it is normally not expressed, reduces biomass accumulation, perhaps through interfering with photosynthetic carbon fixation, which in C4 plants is limited to this compartment (Figures 5 and 7, Table S2, Figure S15). Another possibility is that the excess ZmLOX6 in stomatal guard cells when expressed under the control of p*ZmrbcS* promoter interfered with stomatal behaviour (Figure S14). Transgenic ZmLOX6 was unlikely to affect stomatal behaviour directly, however, when it was expressed under the control of the p*ZmPEPC* promoter, which did not express in the guard cells (Figures S13 and S14).

Since we did not measure stomatal conductance, it is not known whether an increase in ZmLOX6 activity in correspondence to its higher accumulation in the mesophyll chloroplasts in transgenic plants had any indirect effect on transpiration. It would appear unlikely, however, because increased stomatal conductance would have constrained hybrid performance under water stress. Conversely, a reduction in conductance might have negatively impacted biomass production in the well‐watered plots. The effect of the increased mesophyll ZmLOX6 activity on stomatal conductance, if any, would be expected to be constitutive as the expression of the ZmLOX6 protein in the transgenic events was several‐fold higher than the control plants (Figures 5 and 10). Furthermore, the substrate for ZmLOX6, fatty acid hydroperoxide, must be generated by another plastidial lipoxygenase, ZmLOX10, which possesses both the lipoxygenase and the lyase activities, suggesting that lyase activity is not limiting (Gao *et al*., 2008). It is yet possible that availability of surplus nitrogen during the recovery period following water stress indirectly affected stomatal opening, thus allowing additional carbon fixation that reflected in increased biomass.

Harvest index (percent ratio of grain to total biomass) in maize has remained unchanged around 50% over the last hundred years (Dhugga, 2007). Yield increase over time has thus been realized from an increase in total biomass, which has mainly been accomplished through an increase in planting density (Dhugga, 2007). Increased grain yield in our study presumably resulted from an increase in total biomass, an observation that agrees with the results obtained from the greenhouse seedling assays of transgenic events. In both the greenhouse and field measurements, the expression of the transgene was driven by the mesophyll‐specific p*ZmPEPC* promoter (Figure 10, Table S2b and Figure S15).

We identified a maize lipoxygenase, ZmLOX6, as a vegetative storage protein in mesophyll cells, and elucidated a novel, N‐terminal signal peptide that targeted it to chloroplasts. ZmLOX6 as percentage of total leaf protein responded to amount and source of applied nitrogen, with reduced form of nitrogen causing the strongest induction. Upon transgenic expression under the control of a light‐inducible promoter that is specifically expressed in the mesophyll cells, ZmLOX6, which normally constitutes less than a per cent of the leaf proteins, accumulated to more than 4%, the same level as for the most abundant mesophyll proteins, ZmPEPC and ZmPPDK. Hybrids derived from transgenic events outperformed the control sibling hybrids under drought stress imposed at flowering, providing evidence that additional, dispensable form of nitrogen stored as a protein in the leaves buffered the plants against water‐deficit stress. Furthermore, our study provides experimental evidence for a link between drought stress tolerance and plant nutrition.

## Experimental procedures

## Identification of nitrogen‐induced polypeptides in maize

Maize seedlings from the inbred line A63 were grown in vermiculite in the greenhouse for 2 weeks and irrigated with Hoagland solution containing different forms and concentrations of nitrogen. Different tissues from the plants were then homogenized in a buffered solution (100 mM MOPS, pH 7.0, 1 mM EDTA, 0.1% Triton X100 and 80 mM sucrose) and centrifuged at 1,000 × g for 10 min followed by 100,000 × g for 40 min. Both the pellet and the supernatant were subjected to SDS‐PAGE using 1.5 × 140 × 160 mM discontinuous gels as previously described (Dhugga *et al*., 1988). The induced polypeptide bands from Coomassie‐stained Tris‐glycine‐SDS gels were excised and washed in 500 μl of 100 mM ammonium bicarbonate, then gradually dehydrated after shaking for 1 h each with increasing acetonitrile concentrations (15%, 50% and 100%, v/v). After further drying for 10 min in a speedvac, the gel pieces were rehydrated in 250 μl of 15% acetonitrile in 100 mM ammonium bicarbonate (ACN‐ABC) containing approximately 4 μg trypsin (Roche 1418025) in ice for 1 h. Unabsorbed fluid was aspirated and discarded, 200 μl of fresh ACN‐ABC added and incubated at 37°C for 16 h. After removal of the fluid into a fresh tube, the hydrated gel pieces were washed in increasing acetonitrile concentrations (15%, 50% and 100%), and the fluid collected from all aliquots was pooled. The pooled aspirant was dried completely under vacuum, and the residue re‐dissolved in 20 μl water containing 0.1% (v/v) formic acid. The entire sample was injected into a 1 μl loop and the peptides were subsequently trapped on a polymeric trap column. Reversed‐phase chromatography was performed using a C18 silica column, 75 μm × 100 mm, at a flow rate of 200 nl.min^‐1^ with an acetonitrile gradient of 3%–85% (v/v). A repeating data‐dependent MS experiment was set up on an LCQ Classic quadrupole ion trap mass spectrometer to acquire one full scan MS followed by three MS/MS scans of the most abundant precursor ions for the duration of the run. The acquired data were then searched using Sequest software to assign sequences to the individual peptide fragments.

## Expression pattern of *ZmLOX6* by RNA‐seq

The expression pattern of *ZmLOX6* gene in different tissues was compiled from the in‐house RNA‐Seq database as described previously (Shi *et al*., 2015).

## Expression of recombinant ZmLOX6, ZmPEPC and ZmPPDK proteins in E. coli and antibody production

Full‐length coding sequence of *ZmLOX6*, *ZmPEPC* and *ZmPPDK* was amplified by PCR from respective expressed sequence tag (EST) clones obtained from the DuPont Pioneer (now Corteva Agriscience) EST archive by PCR to generate fragments that were in‐frame with HIS tag in pET‐28a (Novagen) vector. In‐frame fusions were confirmed by sequencing. Respective pET‐28a‐*gene* vectors were transformed into expression host Rosetta (DE3) pLact (Novagen) cells using the supplier's standard protocol. Cell lysis and solubilization were achieved using the following detergent lysis buffer: 50 mM sodium phosphate pH 7.7, 2% (w/v) Triton X‐100 and +/‐ 200 μg/mL lysozyme. Recombinant ZmLOX6 protein was found to accumulate in the insoluble inclusion bodies and was only partially liberated from this fraction with 8 M urea.

To overcome the solubility problem, bacteria from a single colony obtained from a freshly streaked plate were used to inoculate 100 mL of LB medium containing appropriate antibiotic(s) for the plasmid and host strain. After incubating overnight with shaking at 37°C, 20 mL culture was added to 500 mL of LB medium with appropriate antibiotic(s) at 37°C until OD_600_ reached 0.5. After adding IPTG to a concentration of 1 mM, the temperature was reduced to 24°C and incubation continued with shaking for another 4 h. The broth was centrifuged at 14,000 xg for 10 min and the bacterial pellet dissolved in Novagen 1X Ni‐NTA binding buffer (50 mM sodium phosphate, pH 8.0, 300 mM NaCl and 10 mM imidazole) containing 1 mM PMSF, 10 µ M leupeptin and a complete protease inhibitor cocktail tablet (Roche Cat. No. 11873580001). After sonication, the suspension was centrifuged at 14,000 xg for 30 min and the supernatant applied to a Ni‐NTA column equilibrated with binding buffer. Ten column volumes of wash buffer (50 mM sodium phosphate pH 8.0, 300 mM NaCl and 20 mM imidazole) were passed through the column. The recombinant protein was eluted with elution buffer (50 mM sodium phosphate pH 8.0, 300mM NaCl and 250 mM imidazole). One mL fractions were collected and run on an SDS‐PAGE gel to visualize the recombinant protein.

The recombinant protein (~2 mg) was injected into rabbits to raise antisera as previously described (Dhugga and Ray, 1994) at Strategic Biosolutions (www.strategicbiosolutions.com). Rabbits were bled after the third booster injection. The antibody so generated recognized a single polypeptide band of ~100 kDa on protein blots of maize leaf extracts.

## High‐throughput protein extractions from maize leaf samples for ELISA

Six, 6 mM leaf punches were collected in megatitre tubes and quickly frozen in liquid nitrogen. To each tube, two stainless steel beads were added followed by 400 µI of protein extraction buffer consisting of 100 mM Hepes pH 7.5, 20% (v/v) glycerol, 1 mM EDTA, 1 mM EGTA, 0.1% (v/v) Triton X‐100, 1 mM benzamidine, 1 mM 6‐aminohexanoic acid, 1 mM phenyomethylsulfonyl fluoride, 10 µ M leupeptin and 1 mM dithiothreitol. Samples were ground twice in a Genogrinder instrument (Geno/Grinder 2000 from BT&C/OPS Diagnostics, 672 Rt., 202‐206 North Bridgewater, NJ) at 1 × 700 setting for 30 s. After centrifuging the megatitre rack at 4000 rpm in an Avanti JS 5.9 rotor for 15 min at 4°C, the supernatant was collected into a second 96‐well format megatitre rack and quickly frozen in liquid nitrogen. Protein concentration was measured using assay kit from Pierce (Pierce Chemical Company, Rockford, IL). Average protein concentration in the extract was 3 mg/mL.

## Development of ELISA for ZmLOX6, ZmPEPC and ZmPPDK proteins

All the reagents were prepared in deionized water. The leaf extract (50 μl) was diluted to 500 µ l with Tris‐buffered saline (TBS) solution (50 mM Tris‐CI, pH 9.0 and 150 mM NaCl), 50 µ l of the diluted extract added to each well in a 96‐well microtitre plate and incubated at 37°C for 2 h to overnight at room temperature. After poor binding of the proteins to the wells was observed at pH 8, we tried several pH and salt concentration variations before settling on pH 9. The plates were rinsed with deionized water 3 × 1 min followed by the addition of 200 µ l blocking buffer (TBS containing 0.3% (w/w) Triton X‐100 and 0.25% (w/w) BSA). After incubation for 30 min at RT, the plates were washed with distilled water 3 × 1 min. Primary anti‐ZmLOX6 antibody solution (50 µ l) diluted in blocking buffer was added to each well and incubated for 2 h at RT. Following 3 × 1 min washes with water, 50 μl secondary antibody solution (1:25,000 dilution of goat anti‐rabbit IgG coupled to alkaline phosphatase; Sigma A3687) in blocking buffer was added to each well, and incubated for 2 h at RT. The plates were washed three times in water, filled with TBST and incubated 10 min. After discarding TBST, the plates were washed 3 × 1 min in water followed by the addition of 75 μl of substrate solution containing 10% (w/w) diethanolamine, pH 9.8, 0.5 mM MgCl_2_ and 0.01% (w/w) thimerosal (Sigma T5125) to each well and incubated for 1 h at RT in the dark. The reaction was stopped with 25 μl 0.5 M NaOH solution and absorbance measured at 405 nm.

Respective calibration curves to predict protein concentrations from absorbance readings were developed by spiking the leaf extracts with sequentially higher concentrations of the expressed, purified proteins followed by ELISA assays.

## Western Analysis of ZmLox6

Total leaf protein (~10 µ g per lane) was loaded onto an SDS‐PAGE gel (BioRad Tris‐HCl, 4%–15% gradient criterion gels, cat. no. 3450024) and, after electrophoresis, proteins transferred to a polyvinylidine fluoride (PVDF) membrane, and the membrane incubated for 30 min at RT in blocking buffer followed by incubation with the ani‐ZmLOX6 antibody (1:50,000 dilution in blocking buffer) for 45 min. Membrane was washed with water 3 × 1 min followed by incubation in TBST for 5 min. Secondary antibody solution (1:25,000 dilution of goat anti‐rabbit IgG conjugated to alkaline phosphatase from Sigma, A3687, in blocking buffer) was added followed by incubation for 45 min. After washing in water 3 × 1 min, the membrane was incubated with blocking buffer for 5 min. Immun‐StarTM AP chemiluminescence from the Bio‐Rad kit was added and the membrane was exposed to an X‐ray film in the dark.

## Immunopurification of native ZmLOX6 protein from maize leaves

Thermo Scientific Pierce^TM^ Protein‐A IgG plus Orientation kit (catalogue no. 44893) was used as per manufacturer’s instructions to purify the ZmLOX6 protein from leaf extracts. Two mL of ZmLOX6 antibody (43 mg.mL^‐1^) was mixed with 2 mL of antibody binding–washing buffer (50 mM sodium borate, pH 8.2), which was then applied to Protein‐A affinity column equilibrated with the antibody binding–washing buffer. This column was placed on a rolling shaker for 30 min at room temperature, followed by removal of top and bottom caps sequentially and washing with 5 mL binding–washing buffer twice. Crosslinking of the bound antibody was accomplished with the disuccinimidyl substrate solution (13 mg.mL^‐1^ of dimethyl sulphoxide) and then mixed with 1.5 mL of crosslinking buffer (0.1 M phosphate, 0.15 M NaCl, pH 7.2), followed by loading on the affinity column. After incubation at RT for 1 h and washing with 10 mL of binding–washing buffer, the remaining active sites were blocked by adding 2 mL of blocking buffer (0.1 M ethanolamine, pH 8.2). The uncoupled IgG was washed off with 5 mL of IgG elution buffer (0.1 M glycine, pH 2.8), followed by washing sequentially with 10 mL of the antibody binding–washing buffer and then with the binding buffer. Concentrated maize leaf total protein (~40 mg.mL^‐1^) in 4 mL was mixed with 4 mL of binding buffer and agarose resin coupled to anti‐ZmLOX6 antibody and placed on a rolling shaker overnight at 4°C. The agarose resin was then washed with the binding buffer until absorbance at 280 nm reached baseline. The bound protein was eluted in 10 mL elution buffer (0.1 M glycine, pH 2.8). The eluted 1 mL fractions were neutralized with 1 M Tris‐Cl, pH 9.5 and analysed by SDS‐PAGE to determine the purification of the protein. Western blotting was performed to confirm the identity of the ZmLOX6 protein. N‐terminal amino acid sequencing of the PVDF‐bound protein was carried out at the protein sequencing facility of Iowa State University (Ames, IA).

## Generation of fusion protein of the ZmLOX6 chloroplast signal peptide and green fluorescent protein

*Aequorea coerulescens* GFP (*AcGFP*) was from Takara Bio, Mountain View, CA. The nucleotide sequence corresponding to the 60, 62 or 81 N‐terminal amino acids was fused with the AcGFP sequence in separate constructs. Positive clones for in‐frame fusion were confirmed by sequencing. The transcription cassette was driven by the p*ZmUbi‐intron* promoter. These vectors were introduced into maize leaf by particle gun bombardment.

## Transient assays

Maize leaf segments used for the bombardment were generated from seedlings grown hydroponically for 15–20 days from maize kernels sandwiched between two layers of blotting paper placed in a water reservoir. Leaf segments (ca. 30–40 mm in length) were placed abaxial side up on top of agar in a petri dish. Plasmid DNA‐coated 600 nm gold particles (BioRad) were bombarded using a gene gun set at 1100 psi. Leaf segments were examined beginning at 24 h post‐bombardment.

## Immunocytochemistry

Leaves from the maize cultivar Gaspe Flint were cut into 2–3 mm pieces and fixed in 2% paraformaldehyde and 0.5% glutaraldehyde in 100 mM sodium phosphate (pH 6.8) for 3–4 h at room temperature followed by 12 h at 4°C. Following dehydration in a graded ethanol series, the samples were infiltrated in a graded series of LR White resin in ethanol. The individual leaf pieces were polymerized in gelatin capsules for 2 d at 55°C.

For immuno‐based localization of ZmLOX6, 700 nm sections from LR White‐embedded leaves were cut perpendicular to the leaf surface, transferred to Vectabond‐treated (Vector Laboratories Inc., Burlingame CA) cover glasses (No. 1.5) and allowed to dry overnight at 40°C. The cover‐glass with attached sections was treated with 0.05 M glycine in PBS for 30 min followed by blocking in Aurion blocking solution (Aurion Immuno Gold Reagents and Accessories, Wageningen, NL) for 30 min. The sections were then probed with an affinity‐purified rabbit polyclonal antibody against ZmLOX6 diluted to 5 µ g.mL^‐1^ for 2 h at RT. Following rinsing in PBS for immunofluorescence, the sections were treated with an Alexafluor 488‐conjugated goat anti‐rabbit secondary antibody (Life Technologies, Grand Island, NY) diluted 1:50 for 2 h at room temperature. After an additional rinse in PBS, the cover glasses were mounted in a drop of Prolong Gold (Life Technologies).

For electron microscopy, the labelling protocol was the same as that used for immunofluorescence with the exception that instead of a dye‐conjugated secondary antibody, a goat anti‐rabbit secondary antibody complexed to ultra‐small gold (Aurion) was used. Following a final rinse in PBS, the samples were fixed in glutaraldehyde and rinsed thoroughly in water prior to silver enhancement for 2 h at room temperature using Aurion R‐GENT SE‐EM reagents according to manufacturer instructions. Following immunogold localization, the sections were stained with 4% aqueous uranyl acetate and Reynold’s lead citrate, carbon coated and imaged by collecting back‐scattered electrons using a Hitachi S‐4800 field emission scanning electron microscope (Rizzo *et al*., 2016).

## Fluorescence microscopy

Confocal images were acquired on an inverted Zeiss LSM510 META laser scanning confocal microscope (Carl Zeiss Microscopy, Thornwood NY). Immunofluorescence samples were imaged using a 40X (NA 1.3) oil immersion plan apochromat objective lens. Data from the bombardment experiments were acquired using a 40X (NA 1.2) water immersion C‐apochromat objective lens and a configuration designed to capture simultaneously both AcGFP emission and chlorophyll autofluorescence. The configuration utilized a 488 nm argon laser line for excitation and a 500–550 nm emission filter for AcGFP and a 565–615 nm emission filter for chlorophyll. In some instances, a second configuration was used to image cell wall autofluorescence. This configuration utilized 720 nm multi‐photon excitation and a 390–465 nm emission filter.

## Generation of ZmLOX6 overexpression constructs and transgenic plants

A mutli‐slot Gateway (Invitrogen^TM^) system with several in‐house modifications was used to generate *ZmLOX6* overexpression constructs. Three promoters (*pZmUbi‐intron*, p*ZmrbcS* and p*ZmPEPC*) were cloned individually in Slot 1 vector, whereas PINII terminator was cloned in Slot 3 vector upstream of LTP2‐DsRED‐PINII cassette (in order to use a ‘red‐marker’ for sorting transgenic seeds visually). The coding sequence of *ZmLOX6* was cloned in pENTRY or Slot 2 vector with or without maize ProAleurain (accession X99936.1) signal peptide (SP) and vacuolar targeting signal (VTS) (Griffiths *et al*., 1997). All intermediate vectors were sequenced to ensure quality. The destination vector (pDEST) contained the maize‐optimized phosphinothricin‐N‐acetyltransferase (*MoPAT*) selection marker under the control of p*ZmUbi‐intron* promoter in addition to left border and right border sequences (Dhugga *et al*., 2007). The LR clonase reaction was performed with Slot1, pENTRY, Slot 3 and pDEST vectors. A total of six constructs were generated to drive the overexpression of *ZmLOX6* constitutively (p*ZmUbi intron*), in the bundle sheath cells (p*ZmrbcS*), or in the mesophyll cells (p*ZmPEPC*) with or without the maize ProAleurain signal peptide and vacuolar targeting signal (VTS). The resulting Japan Tobacco (JT) expression vector was quality checked by restriction mapping and PCR and transferred into *Agrobacterium tumefaciens* strain LBA4404 by electroporation. The co‐integrated DNA from *Agrobacterium* was transformed into *E. coli* strain DH10B and the DNA prepared from this was subjected to restriction digestion for quality control. *Agrobacterium*‐mediated transformation of maize immature embryos was carried out as previously described to generate initial T0 events (Zhao *et al*., 2002). All events were characterized at the molecular level by genomic PCR and RT‐PCR. After initial characterization of the transgenic events, a subset of 10 single‐copy events expressing the transgene under the control of the ZmPEPC promoter were advanced to make hybrids for field testing.

## Remobilization of leaf proteins

Sixteen transgenic events and the parental nontransgenic line were grown in the field in Johnston, Iowa, in two replications. Starting at flowering, eight plants were samples for the ear leaf tissue from each of the lines at 6‐ to 8‐day intervals all the way to maturity except between 22 and 37 days after flowering. Protein extracts from each of the samples were subjected to ELISA using the anti‐ZmLOX6 and ZmPEPC antibodies.

## Maize hybrid yield testing and statistical analysis

To evaluate the effect of p*ZmPEPC*::*ZmLOX6* construct on grain yield, field trials were conducted in North America in 2008 and 2009. Transformation of the p*ZmPEPC*::*ZmLOX6* construct into an elite inbred line, PH17AW, produced random insertions or events that were heterozygous for the transgene. Single‐gene insertions were identified by quantitative PCR. Top crosses of those individual event lines onto an elite tester PH176Z2 resulted in F1 hybrid seed that segregated one to one for the transgene. Using an expressed dominant seed coat colour marker, DSRED, F1 seeds positive for the transgene were separated from nontransgenic seeds. The resulting nontransgenic seed lots across all events were bulked together to use as a null comparator to the transgene positive events.

Two unique field environments were established at research centres in Woodland, CA, and Johnston, IA, in 2008. In the Woodland location, where water status is precisely managed, subsurface drip irrigation was used to impose water‐limited conditions to achieve a unique stress treatment (flowering stress or FS). In the year 2009, the experiment was repeated in the Woodland location under both optimal and FS conditions. Drip tape was used to apply irrigation in order to achieve two different treatments. For flowering stress treatment, irrigation was withheld approximately 2 weeks prior to anthesis in order to apply a drought stress through the silk exertion period and then irrigation was resumed 2 weeks after flowering. Total rainfall was 0.9 mm in 2008 and 16.6 mm in 2009. Well‐watered treatments had sufficient irrigation applied in order to avoid any level of drought stress. Each irrigation cycle supplied 13–45 mm of water, which was determined based on the combined soil water content and meteorological data as described in detail in another study (Reyes *et al*., 2015). Total irrigation for the flowering stress treatment was 241 mm and 315 mm, respectively, for years 2008 and 2009. For the well‐watered treatment in 2009, irrigation totalled 722 mm. Water was withheld approximately 2 weeks prior to flowering and through the flowering period until 2 weeks after flowering. Leaf rolling was obvious in the FS environment. In Johnston, which is considered to be an optimal environment for grain yield, rainfall in 2008 was 808 mm and irrigation totalled 19 mm. The field experiments were planted in incomplete block designs with 8, 6 and 4 replications for the Woodland flowering stress (FS), Woodland optimal conditions (Opt), and Johnston (Opt) respectively. Experimental entries were grown in four‐row plots ranging from 4.4 to 5.3 m in length separated by a 0.5 m alley. Grain yield of the null comparator line under water stress was approximately 180 bu.ac^‐1^ (approximately 11.25 t.ha^‐1^), which represents roughly a 25% yield reduction from the same hybrids grown under optimal conditions (grain yield 223 bu.ac^‐1^ or 13.9 t.ha^‐1^). In Johnston, IA, plots were managed with full irrigation to achieve optimal yield conditions. Null comparator grain yields averaged 203 bu.ac^‐1^ (~12.7 t.ha^‐1^).

Using a standardized quality control procedure, outlier data points were identified and eliminated before final statistical analysis. Grain weight and moisture content for each experimental entry were measured by harvesting the middle two rows of each four‐row plot using a small‐plot research combine. Yield was standardized within the experiment by adjusting the harvested grain weight of each plot to 15% grain moisture. Analysis was conducted using ASREML, and the yield values for each event are presented as best linear unbiased predictor (BLUP) differences relative to the null comparator (Gilmour and Thompson, 1995). To account for field variability, spatial components of variability such as column, row and incomplete block main effects were included in the model. To address patchy spatial variation in the field, an autoregressive correlation (AR1) structure was fitted in both column and row directions. A pairwise, two‐tailed t test of significance between each of the events and the null comparator was conducted at the P < 0.10 level.

An experiment involving 12 commercial hybrids was planted in Woodland, CA, in 2007, using the aforementioned protocol. Leaf discs from the ear leaf, which was generally 8^th^ aboveground leaf, were collected, frozen and processed as described in earlier sections. ELISA assays were then performed for the ZmLOX6, ZmPEPC and the ZmPPDK proteins. Path coefficient analysis was performed as previously described (Silva *et al*., 2017, 2018; Wright, 1921).

## Seedling nitrogen uptake and utilization assay

Two seeds of each transgenic event driven by p*ZmrbcS* and p*ZmPEPC* were planted in 15‐cm‐diameter pots filled with Turface and grown semi‐hydroponically in a greenhouse (30°C /25°C, day/night) (Loussaert *et al*., 2017). Pots were thinned to one plant after emergence. Nitrate (potassium form) levels were maintained at 5 mM concentration by completely replacing the nutrient medium up to three times a day depending upon the stage of growth. Transgenic events and a bulked construct null were arranged in a completely randomized design with nine replicates per treatment. Plants were harvested at 18 days. After washing Turface, roots were separated from the shoot, both dried at 70°C for 72 h and dry mass measured. Dried roots and shoots were ground separately, and a sample removed for micro‐Kjeldahl analysis. Data were subjected to analysis of variance and event means were compared with construct bulked null means by Student’s t analysis (Loussaert, 1992).

## Conflict of interest

The authors are, or were, scientists employed at Corteva Agriscience, a world leader in developing agricultural products, with the goal to ‘enrich the lives of those who produce and those who consume, ensuring progress for generations to come’. HKRA, LMA, RG and KSD are co‐inventors on pending patent applications covering this work. Materials reported in this paper may be subject to third‐party ownership and/or to governmental regulations. Availability of materials reported in this paper to academic investigators for noncommercial research purposes under an applicable material transfer agreement will be subject to the requisite permission from any third‐party owners of all or parts of the materials and to governmental regulation considerations. Obtaining the applicable permission from such third‐party owners will be the responsibility of the requestor. Transgenic materials reported in this paper would only be made available if in full accordance with all applicable governmental regulations.

## Author contributions

HKRA overexpressed various proteins, developed and deployed ELISA assays, immunopurified mature ZmLOX6 and obtained N‐terminal sequence, prepared vectors for transit peptide validation and identified additional nitrogen‐inducible proteins. RG validated the promoters, made vector constructs and oversaw molecular characterization of transgenic events. LMA originally identified nitrogen‐induced proteins, prepared tryptic digests, overexpressed ZmLOX6 in coli, raised anti‐ZmLOX6 antibody, and validated it by Western analysis. LPF overexpressed ZmPPDK protein, raised and validated antibodies. JH carried out mass spectroscopy of tryptic digests to identify nitrogen‐inducible proteins. GZ raised antibodies against the ZmPEPC protein and provided the expression vector for ZmPPDK. TMB carried out transient expression assays, immunocytochemical localization and imaging. RJH supervised microscopy work and helped interpret results. JRS tested commercial hybrids under drought stress. DFL performed seedling nitrogen uptake and utilization assays. BW, RLH and SMH produced hybrids of transgenic events, conducted field trials, collected and analysed field data. JEH helped with strategy development for drought tolerance and provided gene expression data. KSD conceptualized and designed the entire study, supervised the project, dissected vascular bundles and mesophyll followed by Western analysis, affinity‐purified ZmLOX6‐specific IgG for microscopy, analysed and interpreted the laboratory and field data, made all the figures and tables and wrote and revised the paper.

## Supporting information


**Figure S1** Maize seedlings grown in vermiculite with various amounts of nitrogen.
**Figure S2** Separated leaves from 16‐day‐old plants, and a corresponding Coomassie bluestained gel of proteins extracted from these leaves.
**Figure S3** Nitrogen‐induced leaf proteins from maize identified by mass spectroscopy.
**Figure S4** Tryptic peptides of ZmLOX6 protein revealed by mass spectroscopy, and signal peptide identified by N‐terminal sequencing of the purified mature protein.
**Figure S5** Protein expression and antibody specificity of anti‐ZmLOX6 antibody.
**Figure S6** ELISA assay development for the ZmLOX6, ZmPEPC, and ZmPPDK proteins.
**Figure S7** Regression of grain yield on the ear leaf proteins at flowering under well‐watered conditions and managed drought stress.
**Figure S8** Path coefficient analysis of the influence of leaf proteins on grain yield under wellwatered conditions and managed drought stress.
**Figure S9** Quantification of the ZmLOX6 protein by ELISA in different zones of a maize leaf from plants grown at different levels of nitrogen.
**Figure S10** Tissue localization of ZmLOX6 after dissection of vascular bundles and mesophyll cells.
**Figure S11** Immunocytochemical localization of ZmLOX6 in a maize leaf and root with light and electron microscopy, respectively.
**Figure S12** Immunopurification of mature ZmLOX6 protein from maize leaf chloroplasts.
**Figure S13** Immunocytochemical localization of ZmLOX6 in a maize leaf expressing the *ZmLOX6* gene driven by the pZ*mPEPC* promoter. Lack of expression in stomata is clear.
**Figure S14** Micrographs from transgenic maize leaves overexpressing ZmLOX6 under the control of the p*ZmPEPC* and p*ZmrbcS* promoters.
**Figure S15** Seedling growth of transgenic events expressing *ZmLOX6* under the control of p*ZmPEPC* and p*ZmrbcS* promoters.
**Table S1** Agronomic traits for the field‐grown transgenic maize hybrids overexpressing ZmLOX6.
**Table S2** Seedling growth assay of transgenic maize events expressing *ZmLOX6* gene under the control of the p*ZmPEPC* and p*ZmrbcS* promoters.

## Data Availability

All data supporting the findings of this study are available in the article and its Supplemental Information files. Plasmids and antibodies used in this study can be provided under an applicable material transfer agreement to academic investigators for noncommercial research.
