# Supplementary material for: A vegetative storage protein improves drought tolerance in maize
Source: Plant Biotechnol J. 2021 Dec 8;20(2):374–89. doi: 10.1111/pbi.13720 (PMC8753363; doi:10.1111/pbi.13720)
Supplement: Supplementary file 1 — Figure S1 Maize seedlings grown in vermiculite with various amounts of nitrogen. Figure S2 Separated leaves from 16‐day‐old plants, and a corresponding Coomassie bluestained gel of proteins extracted from these leaves. Figure S3 Nitrogen‐induced leaf proteins from maize identified by mass spectroscopy. Figure S4 Tryptic peptides of ZmLOX6 protein revealed by mass spectroscopy, and signal peptide identified by N‐terminal sequencing of the purified mature protein. Figure S5 Protein expression and antibody specificity of anti‐ZmLOX6 antibody. Figure S6 ELISA assay development for the ZmLOX6, ZmPEPC, and ZmPPDK proteins. Figure S7 Regression of grain yield on the ear leaf proteins at flowering under well‐watered conditions and managed drought stress. Figure S8 Path coefficient analysis of the influence of leaf proteins on grain yield under wellwatered conditions and managed drought stress. Figure S9 Quantification of the ZmLOX6 protein by ELISA in different zones of a maize leaf from plants grown at different levels of nitrogen. Figure S10 Tissue localization of ZmLOX6 after dissection of vascular bundles and mesophyll cells. Figure S11 Immunocytochemical localization of ZmLOX6 in a maize leaf and root with light and electron microscopy, respectively. Figure S12 Immunopurification of mature ZmLOX6 protein from maize leaf chloroplasts. Figure S13 Immunocytochemical localization of ZmLOX6 in a maize leaf expressing the ZmLOX6 gene driven by the pZmPEPC promoter. Lack of expression in stomata is clear. Figure S14 Micrographs from transgenic maize leaves overexpressing ZmLOX6 under the control of the pZmPEPC and pZmrbcS promoters. Figure S15 Seedling growth of transgenic events expressing ZmLOX6 under the control of pZmPEPC and pZmrbcS promoters. Table S1 Agronomic traits for the field‐grown transgenic maize hybrids overexpressing ZmLOX6. Table S2 Seedling growth assay of transgenic maize events expressing ZmLOX6 gene under the control of the pZmPEPC and pZmrbcS promoters. [file PBI-20-374-s001.pdf]

Supporting information

Figure S1. Maize seedlings 11 days after planting. Seeds of maize inbred line A63 were planted in vermiculite and irrigated daily with a Hoagland solution containing either none or various amounts of nitrogen in the form of potassium nitrate or ammonium nitrate. Leaf rolling is obvious when seedlings were irrigated with a solution containing 100 mM potassium nitrate, perhaps because of osmotic stress resulting from excess potassium (c). In contrast, the seedlings look normal when equimolar nitrogen is applied but with ammonium as the counterion instead of potassium (d).

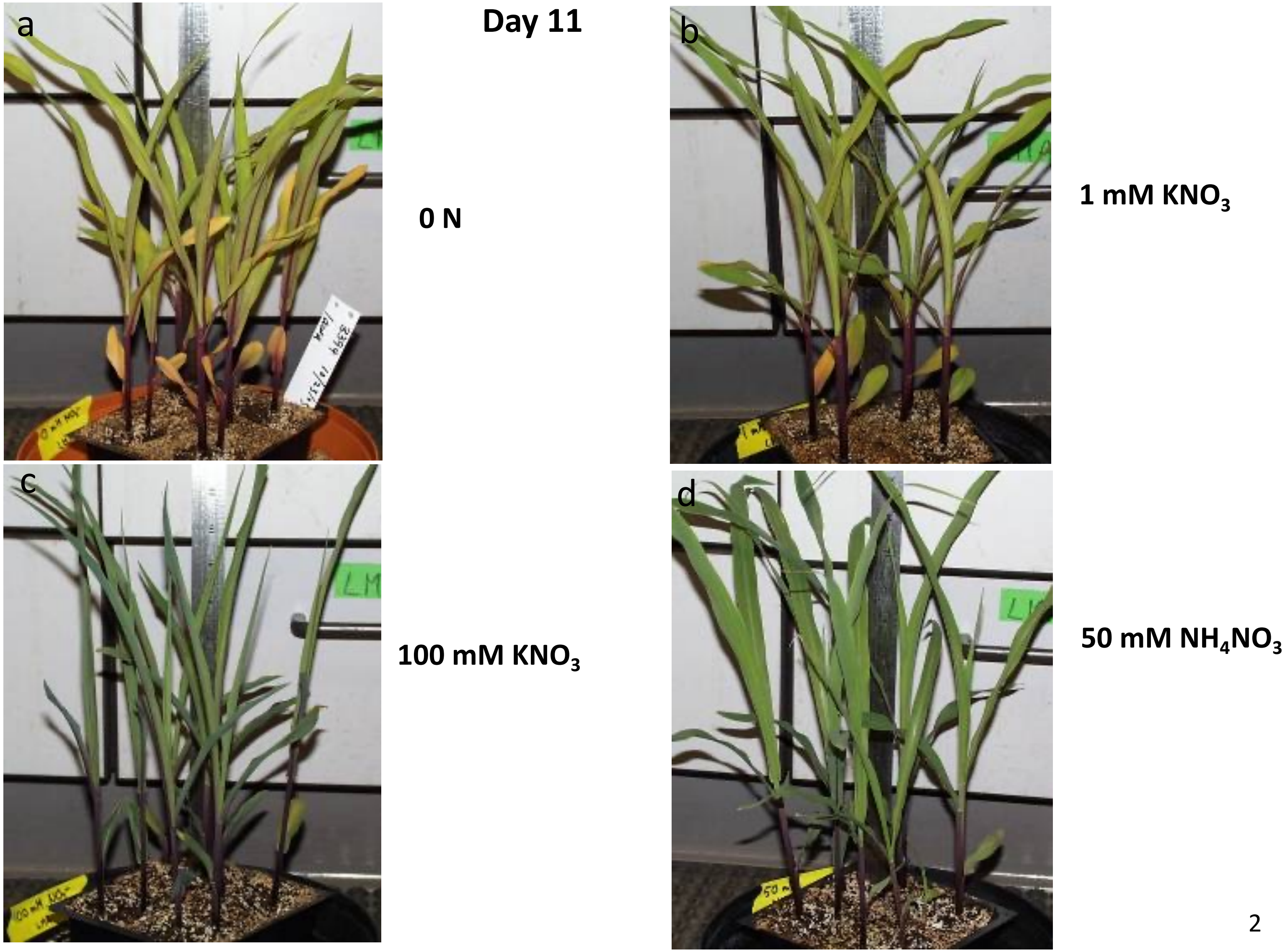

Figure S2. Leaves separated from two 16-day-old plants grown in vermiculite supplemented with 50 mM ammonium nitrate (a). Leaves are numbered starting with the first true leaf above the coleoptilar leaf (labeled as ‘C’ in figure A). Coomassie blue-stained gel (B) of proteins (25 μg per lane) extracted from individual leaves as shown in panel a of plants grown in 0.1 or 50 mM ammonium nitrate in Hoagland's nutrient medium. Bar scale (panel a): 5 cm.

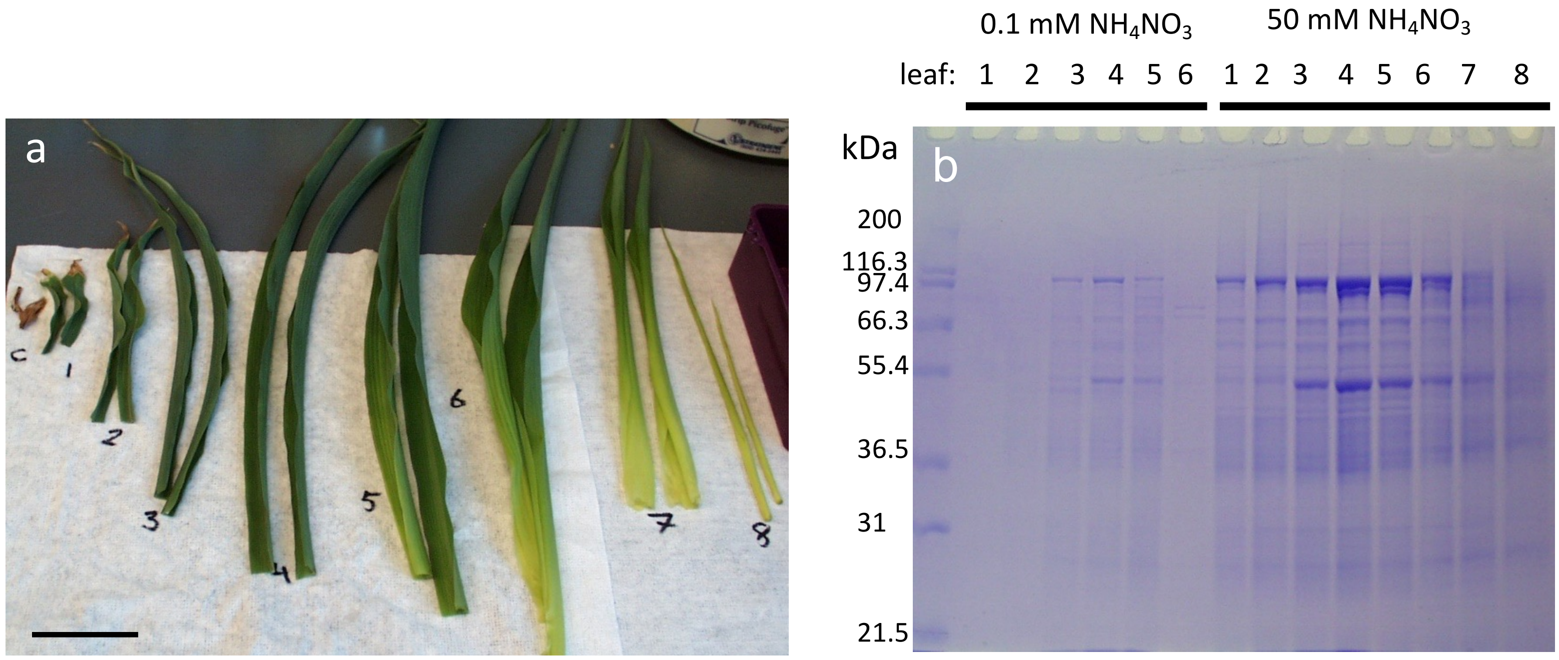

Figure S3. Nitrogen-induced proteins identified by mass spectroscopy from the leaves of maize seedlings. The induced bands were excised, treated with trypsin as described in M&M, and eluted peptides subjected to LC-MS-MS. Five different polypeptides were identified from the 100 kD band and only one from the 50 kD band.

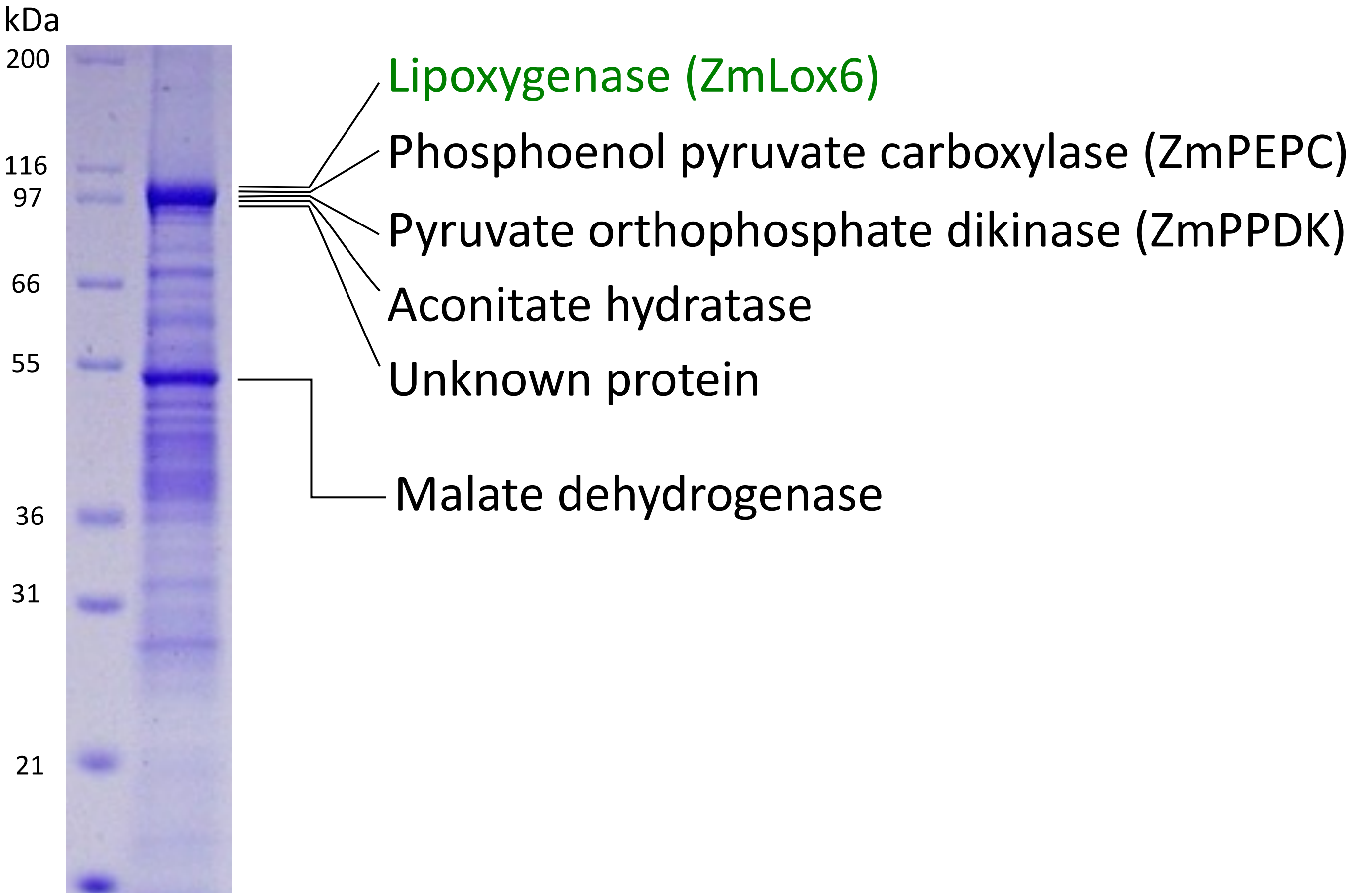

Figure S4 Tryptic peptides revealed by mass spectroscopy corresponding to ZmLOX6 protein (a) are highlighted along with their amino acid positions in the protein in grey boxes in the bar graph below (b). The green arrow (position 1-62) represents the transit peptide (corresponding bold font in the sequence) and blue box represents the fourteen amino acids (blue italics) identified by N-terminal sequencing of the ZmLOX6 protein immunopurified from maize leaves. Potential trypsin target sites in the N-terminal portion of the predicted protein are highlighted in red (Arg) and green (Lys) fonts. Lysyl residue at position 81, where the first trypic peptide was identified, is highlighted. Peptides 2, 4, and 11 contained 1, 2, and 4 additional sites for trypsin digestion, which each resulted in independent peptides as identified by mass spectroscopy but are subsumed in the numbered peptides. A total of 19 peptides were identified. ZmLOX6 amino acid composition in terms of percent frequency and mol percentage of each amino acid in the protein is shown in panel c. The ZmLOX6 protein (GenBank accession DQ335764) has a molecular mass of 97.395 kDa and an isoelectric point of 5.48.

a ZmLOX6 amino acid sequence (total length – 892 amino acids)

```
  1 MMQQLRHSQP SPCLCGLRAA RPMLALGAAA SRSRPAGKLQ PSVCLGLGHV APAAARGQPR
 61 PRAVADSALG ASPTSVHVGG KLLLQNFAAD SQQRLKLSIQ LVSATVADPD GRGVKAEASV
121 LDAVVGSGDS ELDVDLIWDE ALGAPGAVVV KNHSDFPVYL RLLSVPAGVG GADDEAAAVH
181 FACNGWVYPV DKHPYRLFFT NDACVKEETP SALLKYREDE LGALRGDGET TERPFQPWDR
241 VYDYALYNDL GNPDLRQDLA RPVLGGSQEY PYPRRTKTGR PAAKTDPRSE SRAPLDEEIY
301 VPCDERVGFA SIPAPTLPPL GGHFRSLADV YRLFGLDDLG RLPEAKAVIN SGAPFPVVPQ
361 VISVNPTHWR KDEEFARQMI AGANPVCIKR VTKFPLASEL DRGVFGDQDS KITKDHVEKN
421 MGGMTVQQAV EEGRLYVVDH HDWVMPYLKR INELPASEEK AEVSQRKVYA ARTLLFLDGE
481 DSSMLRPLAI ELSSPHPEKE QLGAVSTVYT PPDSGDDGIT AGRFSTWELA KVYASANDAA
541 ENNFVTHWLN THASMEPIVI AANRQLSVLH PIHRLLKPHF RKTLHINAVA RQIIVGSGDQ
601 RKDGSVFRGI DEVTYFPSKY NMEMSSKAYK AWNFTDLALP NDLIKRGLAK GDPKKPETVE
661 LAIKDYPYAV DGLDMWAAIK KWVADYCAIY YADDGAVARD SELQGWWSEV RNVGHGDLAD
721 APWWPAMDCV ADLVETCATV VWLSSAYHAS ISFGQYDYLG FVPNGPSITT RPVPGPDAGA
781 EVTESDFLAS VTPVTEALGF MSIASGPMGL KGTEVYLGQR PDTEQWTRER RAAEALAEFR
841 ARLEEVAGNI DRRNADPALK NRTGQVEVPY TLLKPTAQPG LVLRGIPNSI TV
```

b

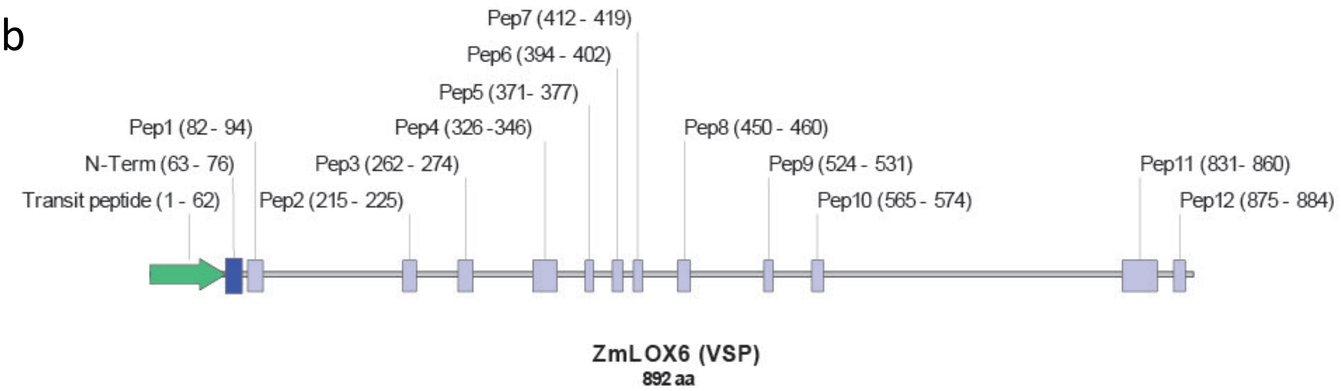

c

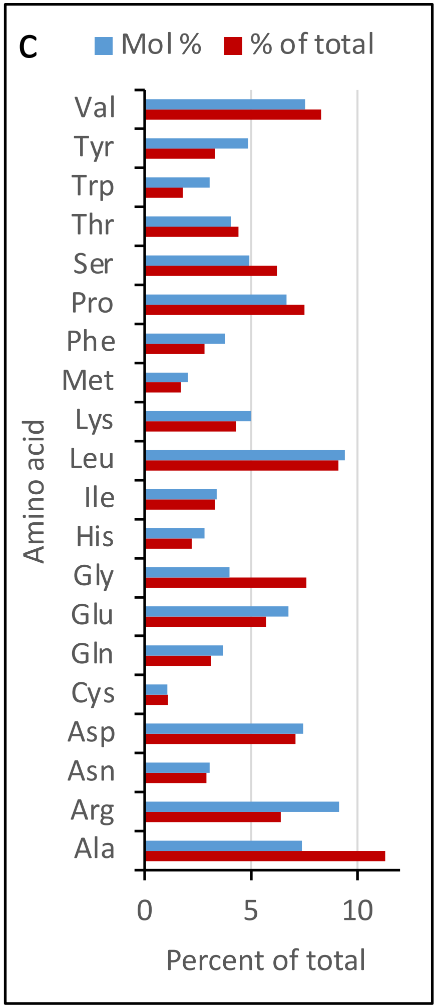

Figure S5 Protein expression, purification and antibody specificity of anti-ZmLOX6 antibody. Purified proteins from expression in *E. coli* (a); lane 1, molecular mass standards, lane 2, ZmLOX6 protein, and lane 3, ZmPEPC protein. Western blot of leaf proteins from the line A63 probed with anti-ZmLOX6 antibody (b). Three lanes contained proteins from leaves 1-3 from the oldest fully expanded leaf upwards at V6 stage.

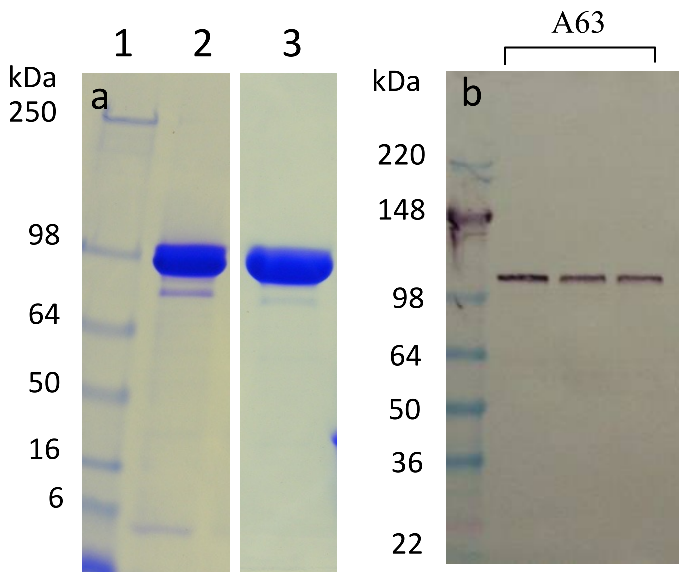

Figure S6 ELISA assay development for the ZmLOX6 (a), ZmPEPC (b), and ZmPPDK (c) proteins. Dividing the numerators in parentheses with the values on the X-axis gives actual dilution factor for the respective antibody. For example, 0.5 represents a 50,000-fold dilution and 2 a 5,000-fold dilution of the anti-ZmLOX6 antibody.

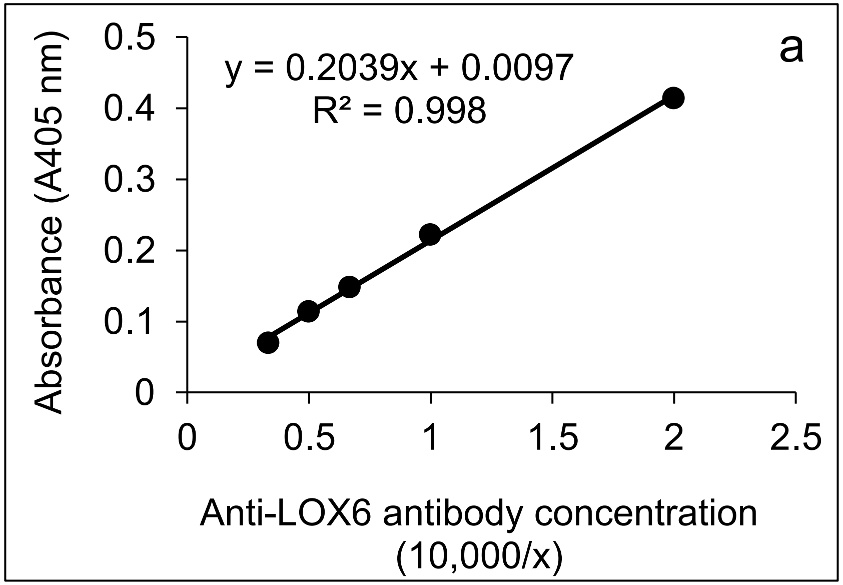

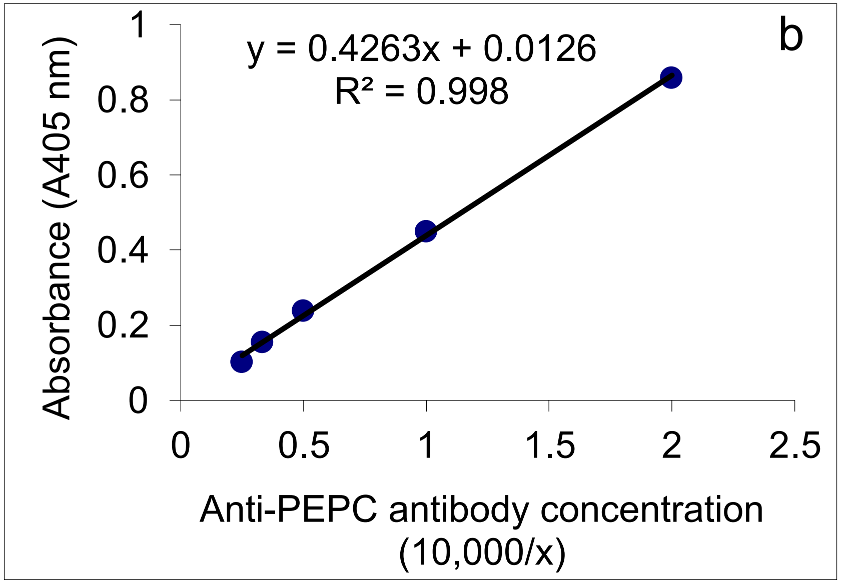

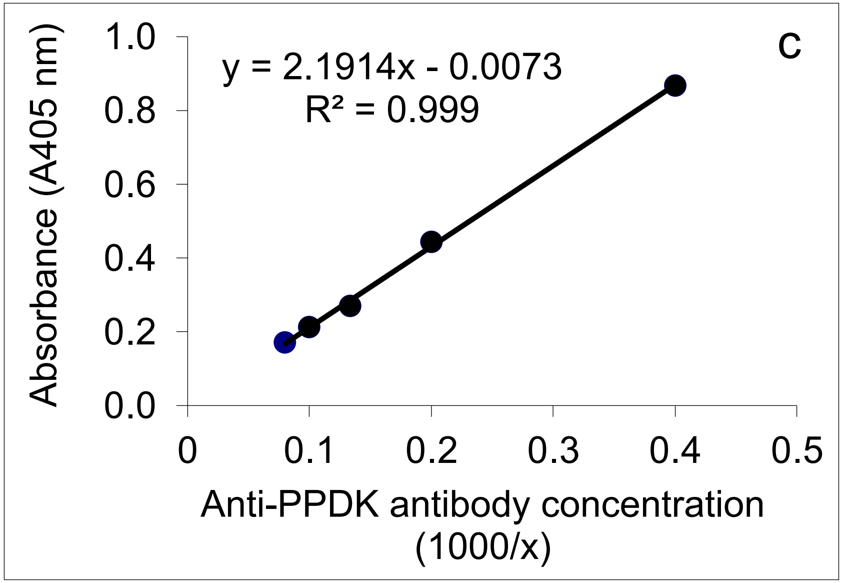

Figure S7 Regression of grain yield on the ear leaf proteins at flowering under well-watered conditions (a, c, e) and managed water stress (b, d, f). Average concentrations of the proteins were: ZmLOX6, 0.7%; ZmPEPC, 4.7%; and ZmPPDK, 4.9%. Under well-watered conditions, PEPC explained most variation, a result that agrees with the other unpublished data from our group. Under stress, however, the LOX6 protein alone explained 56% of the variation. A positive correlation suggests that the hybrids that accumulated more LOX6 protein in their leaves at flowering also had higher grain yield. Although the correlations are relatively weak, an opposite trend was observed with the PEPC and PPDK proteins. Horizontal and vertical bars within the plots are least significant difference (LSD) estimates at 5% level of significance for the traits represented on the respective axes.

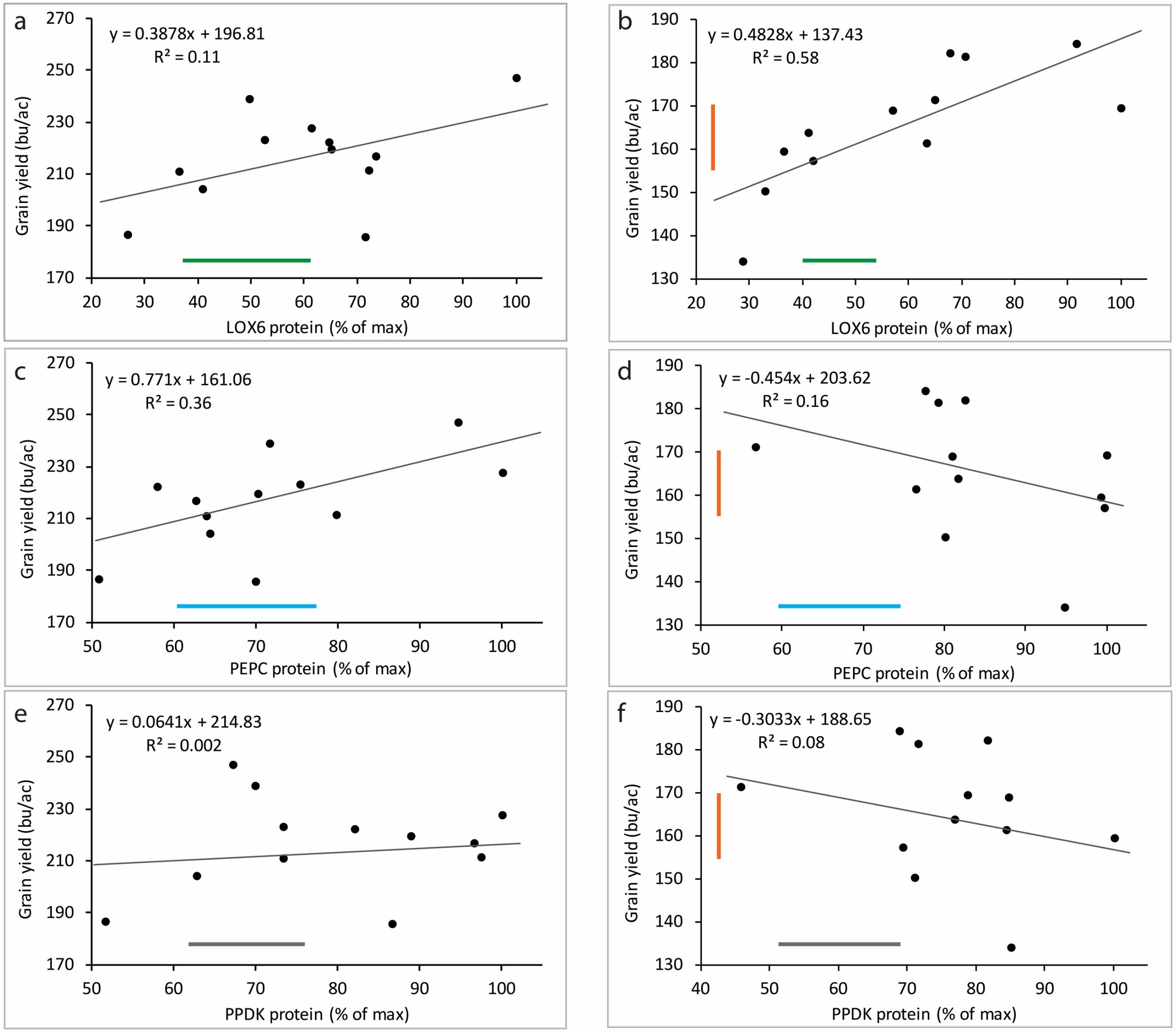

Figure S8 Path coefficients representing direct and indirect contributions of leaf proteins to grain yield under well-watered conditions (a) and managed water stress (b). Above, path diagrams; below, total variation explained in grain yield and variation explained by each protein. Path coefficients are represented by single-headed arrows. Double-headed arrows are for the correlation coefficients between the two variables they connect. Each path coefficient represents the direct and indirect contribution through the other variables it is connected to. Under well-watered conditions, the total variation in grain yield explained (R^2) by the three proteins was 42%, with ZmPEPC explaining the most variation, 30%, a result that is supported by the other, unpublished data from our group on larger germplasm screening. Under water stress, the ZmLOX6 protein alone explained more than half of the variation in grain yield whereas all three proteins together explained 2/3rd of the variation. ZmLOX6 was the best predictor of grain yield under water stress. Further, ZmPEPC and ZmPPDK are the C4 enzymes so altering their expression had the potential to interfere with the seminal process of photosynthesis. ZmLOX6 protein was thus a natural choice for further, in-depth exploration.

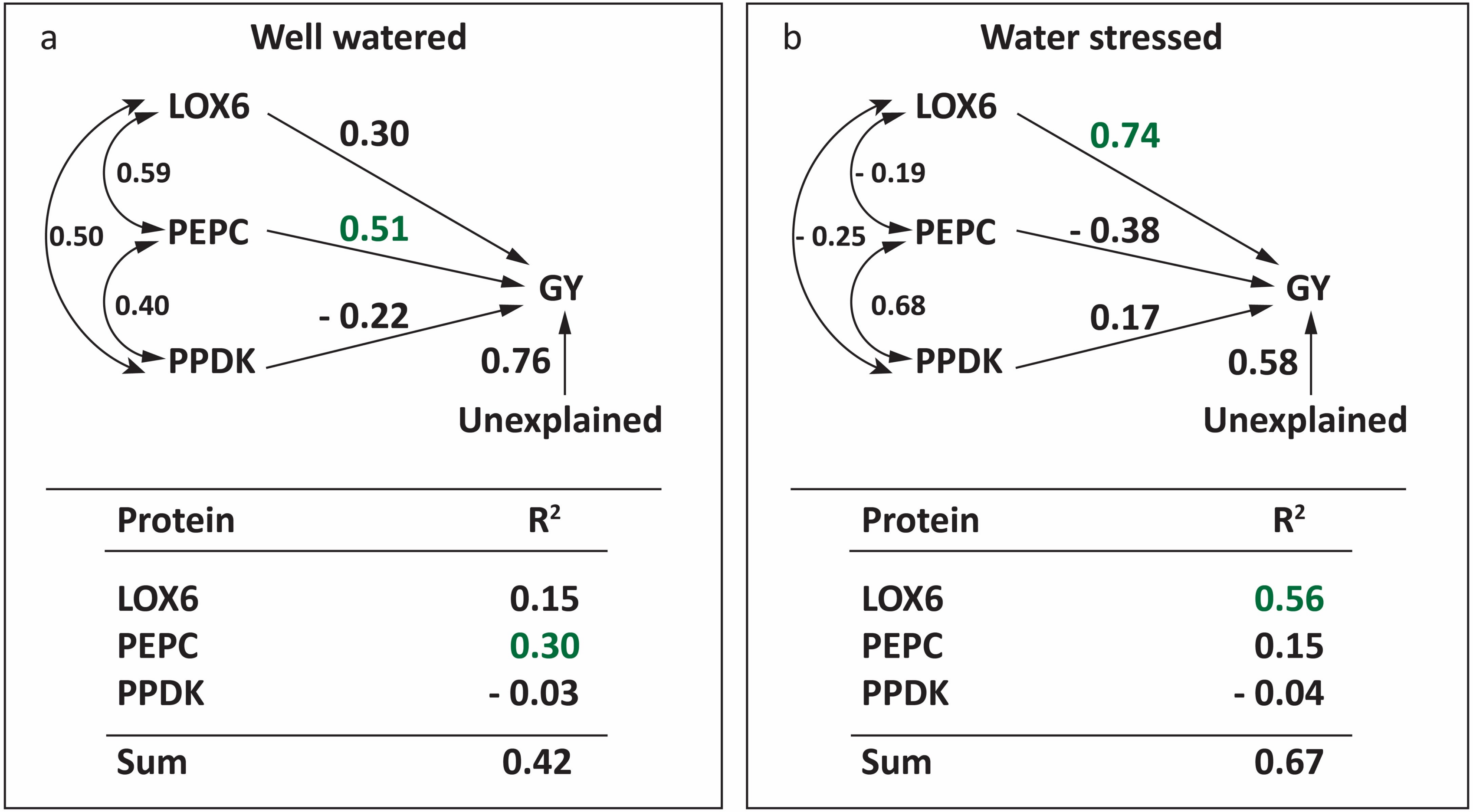

Figure S9 Expression of the ZmLOX6 protein in different sections of the maize leaves (a) collected from the plants grown in different amounts of nitrogen (b) . The ZmLOX6 protein was quantified with an ELISA assay. Bar scale:(right panel) 5 cm.

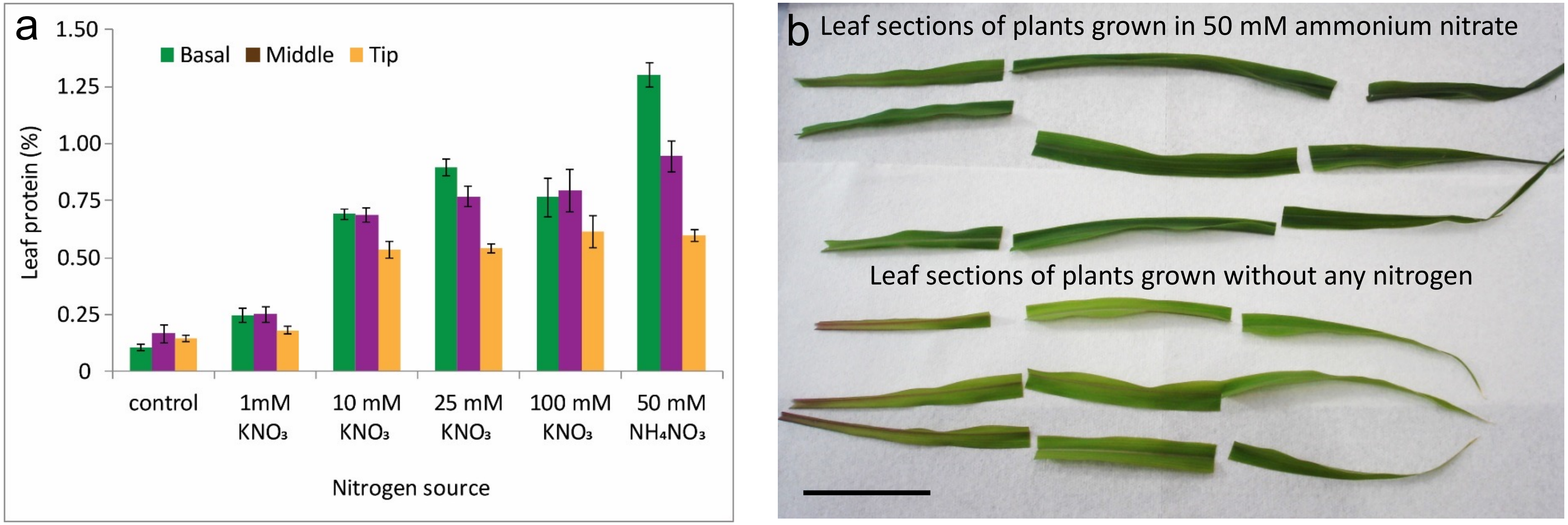

Figure S10 Dissection of vascular bundles and mesophyll from the leaf sheath of a maize plant (a), bundles with attached mesophyll (b), dissected mesophyll (c), and severed vascular bundles (c). Silver-stained SDS gel (e) and western blot probed with anti-ZmLOX6 antibody (f). VB, vascular bundles; MC, mesophyll cells; MW, molecular mass markers. An arrow points to the ZmLOX6 protein. Silver stain is known not to stain some proteins quantitatively and is displayed only to show that approximately equal amount of protein was loaded onto each lane. Bar scale: a, 2 mm; b-d, 1 cm.

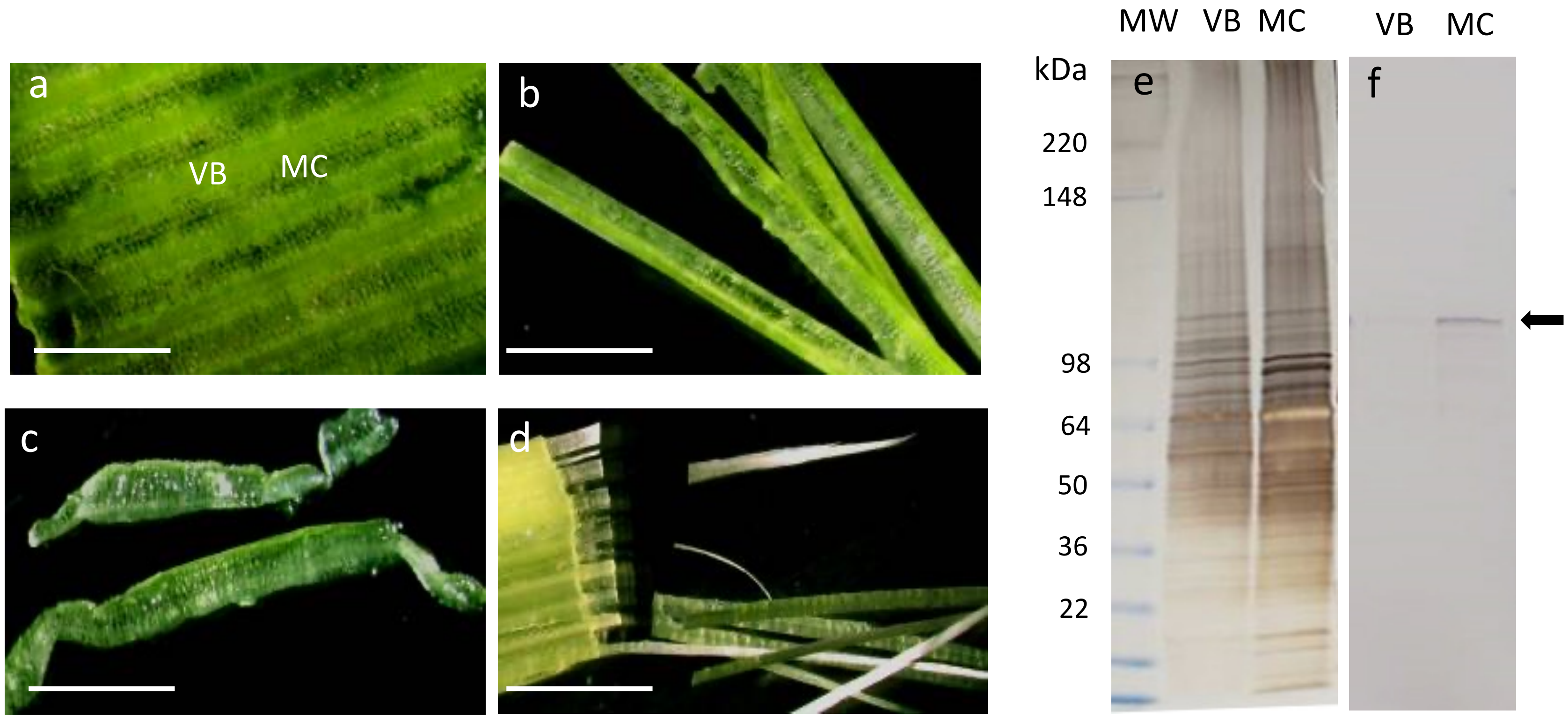

Figure S11 Immunocytochemical localization with ant-ZmLOX6 antibody in a wild type maize leaf with light microscopy (a) and seedling root using electron microscopy (b). ZmLOX6 was localized to mesophyll chloroplasts in the leaf and amyloplasts containing starch granules (S) in the root. Bar scale: a, 20 μm; b, 1 μm.

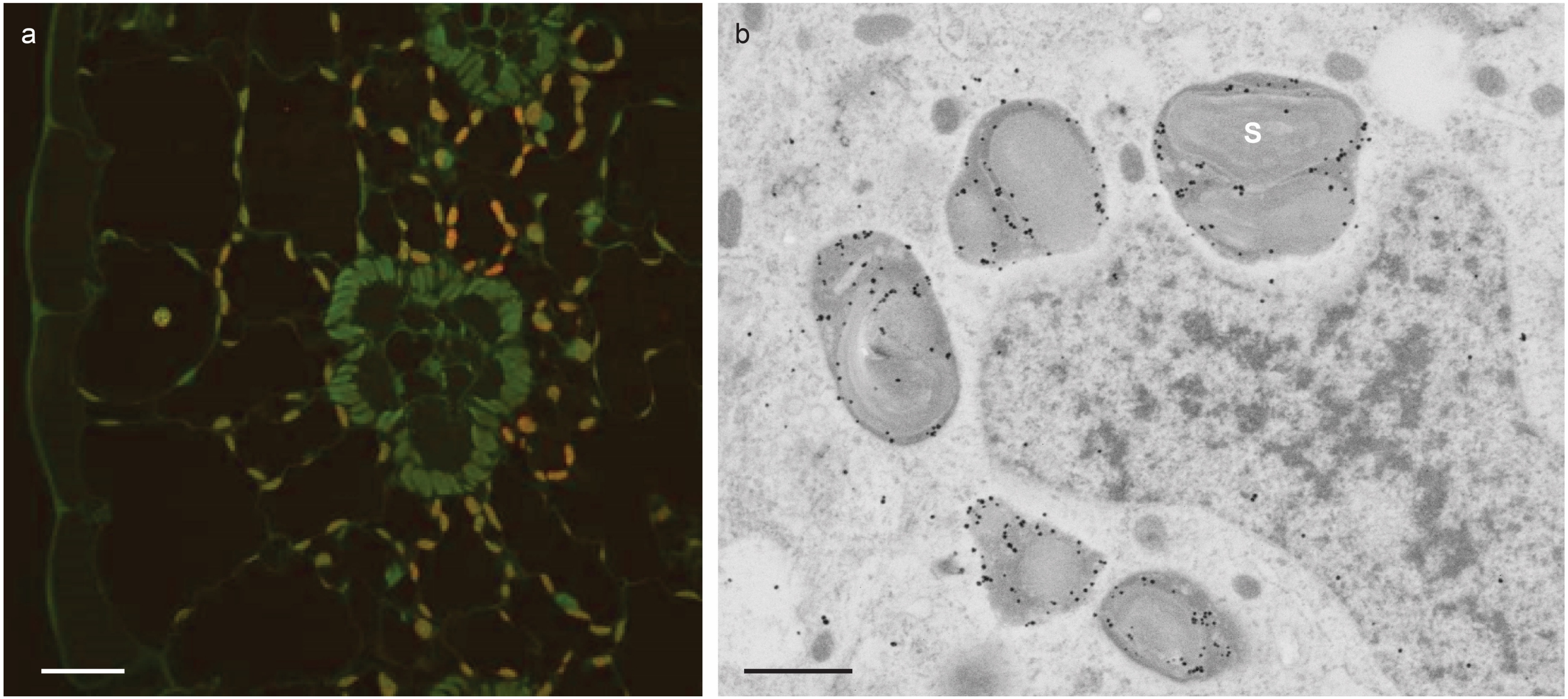

Figure S12 Immunopurification of mature ZmLOX6 protein from maize leaf chloroplasts expressing the *ZmLOX6* gene under the control of the p*ZmPEPC* promoter. Lanes A, flow-through fraction; B to G, wash fractions; H, eluent from 100 mM glycine, pH 2.8; I, eluent from 6M guanidine-HCl wash following glycine elution; and J, eluent from untreated beads from affinity column with 100 mM glycine, pH 2.8. Arrow points to the ZmLOX6 protein.

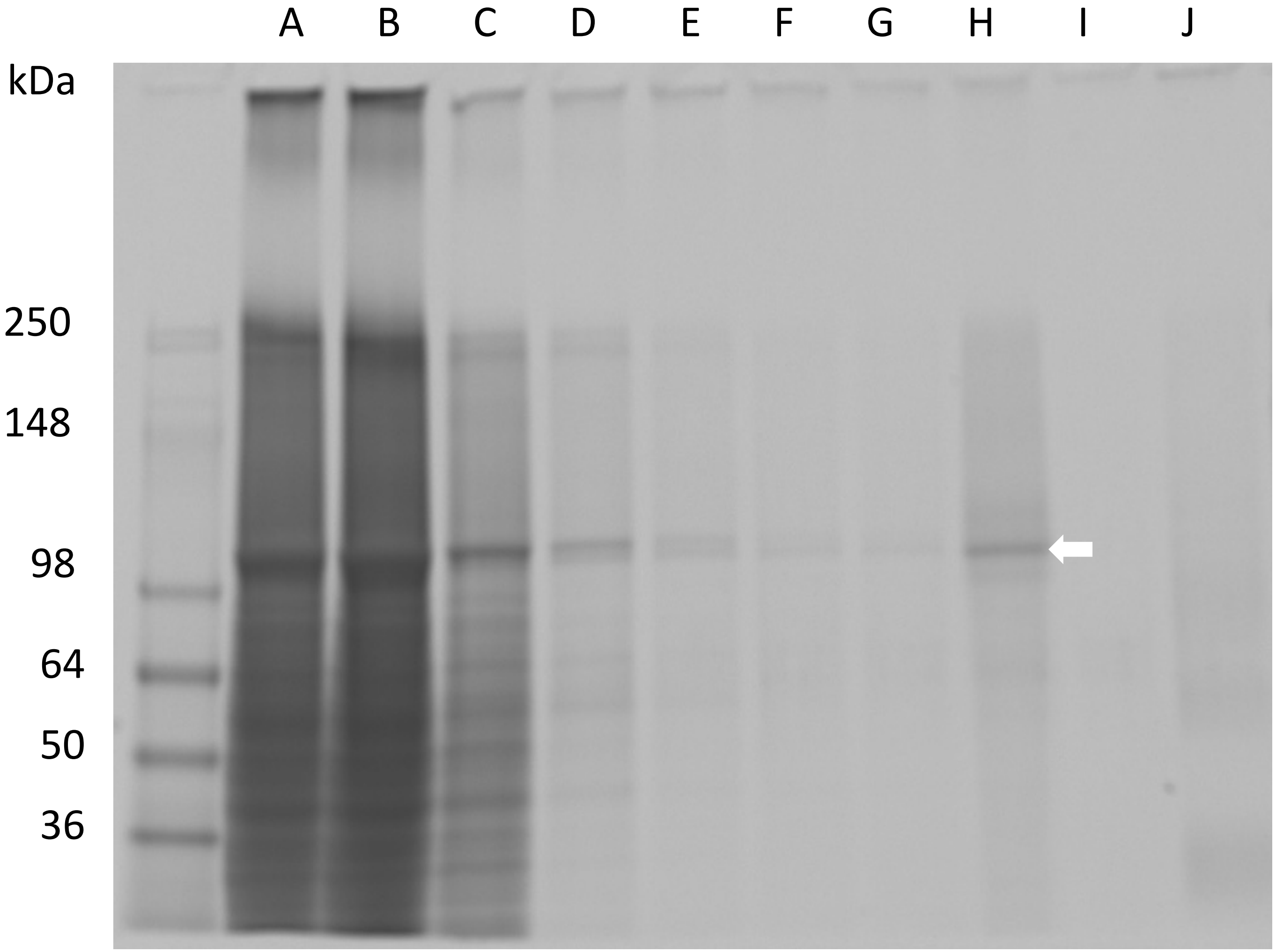

Figure S13 Immunocytochemical localization of ZmLOX6 in a wild type maize leaf (a) and transgenic plants generated with *ZmLOX6* driven by the pZ*mPEPC* promoter (b-d). Panel B is the same as panel c in Fig. 5 and is included as a reference. Stomatal guard cell pairs are pointed out with asterisks. Color was artificially enhanced in panel d. The promoter pZ*mPEPC* was specific to the mesophyll cells and did not appear to express in the epidermis or guard cells. It is possible, but appears unlikely, that some ZmLOX6 protein was expressed in the guard cells as this protein is expressed at low level in the mesophyll cells (a) but was below the threshold of detection with microscopy. Bar scale 20 μm.

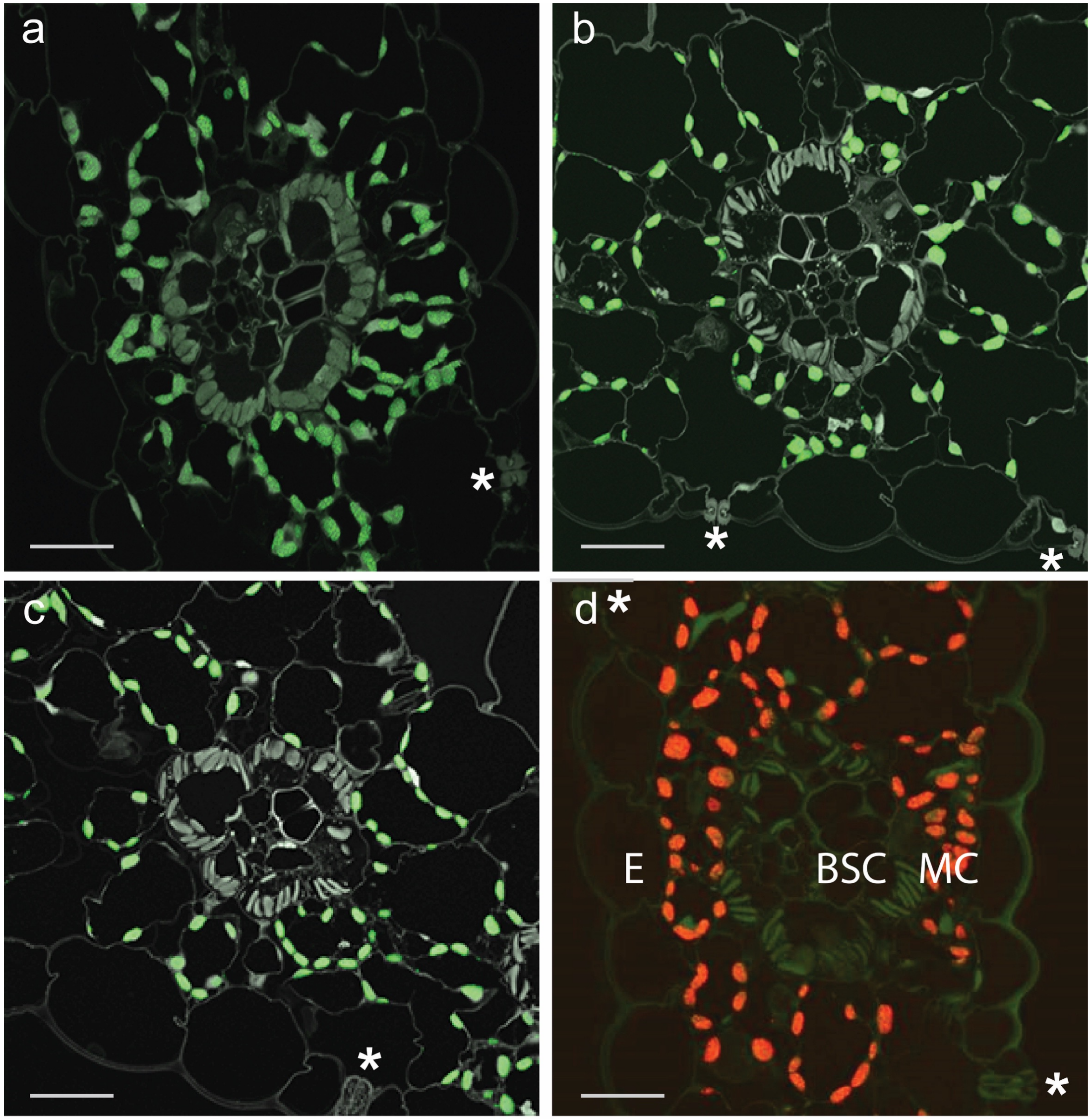

Figure S14 Micrographs obtained from transgenic maize plants overexpressing ZmLOX6 under the control of the p*ZmPEPC* (a) and p*ZmrbcS* (b) promoters. Micrograph 'a' is a truncated, enlarged version of Supplementary Figure 13C. The color in the micrograph is enhanced to ensure detection of any signal in the guard cell pair. p*ZmPEPC* promoter did not appear to express in the guard cells whereas p*ZmrbcS* promoter did. Asterisks point to a guard cell pair in each micrograph. The AcGFP protein in Fig. 3 was expressed in the guard cells because those micrographs were obtained from transient expression of AcGFP under the control of p*ZmUbi-intron* promoter after biolistic transformation, which directly transformed the guard cells. The objective of that experiment was to demonstrate the targeting of the AcGFP protein to the chloroplasts by the novel transit peptide of 62 N-terminal amino acids of the ZmLOX6 protein we identified by N-terminal sequencing of the immunopurified protein from leaves. The micrographs in this figure and in Supplementary Figure 13, in contrast, show the expression of the respective promoters in different cell types in stable transgenic events. Bar scale: 10 μm.

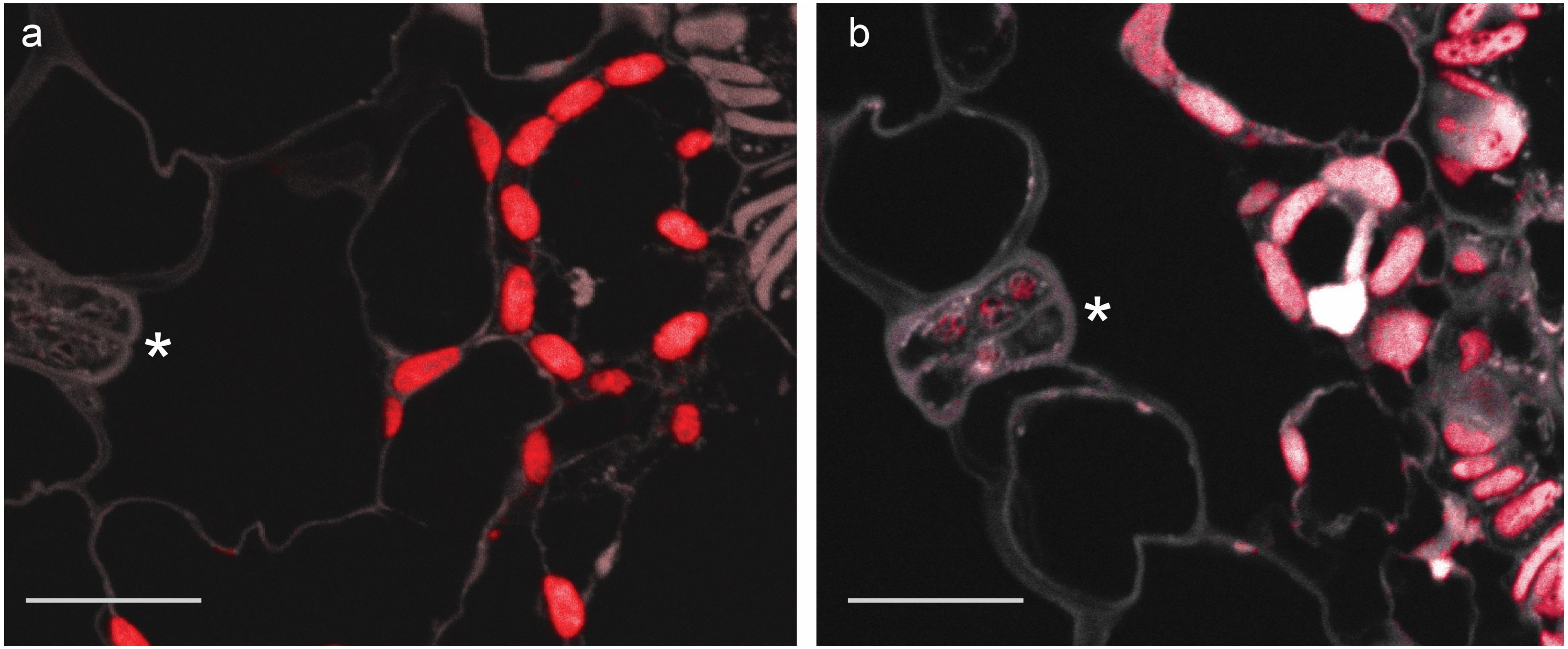

Figure S15 Seedling growth assay of transgenic events expressing *ZmLOX6* gene under the control of a vascular bundle-specific promoter, p*ZmrbcS,* or mesophyll-specific promoter, p*ZmPEPC*. Data from Table 2 were averaged across the transgenic events and controls, respectively, and the ratios of the respective traits in transgenic events against controls presented. The red dotted line represents a ratio of 1 or 100%. Total nitrogen in the transgenic events expressing the Zm*LOX6* gene under the control of the p*ZmPEPC* promoter was increased whereas it was unchanged in the events expressing it under the control of the p*ZmrbcS* promoter. An increase in total biomass explained the increase in total nitrogen in the p*ZmPEPC*-driven events. Expression of the *ZmLOX6* gene under the control of the p*ZmrbcS* promoter increased nitrogen concentration but it was compensated by a reduction in biomass, leaving the total seedling nitrogen unchanged.

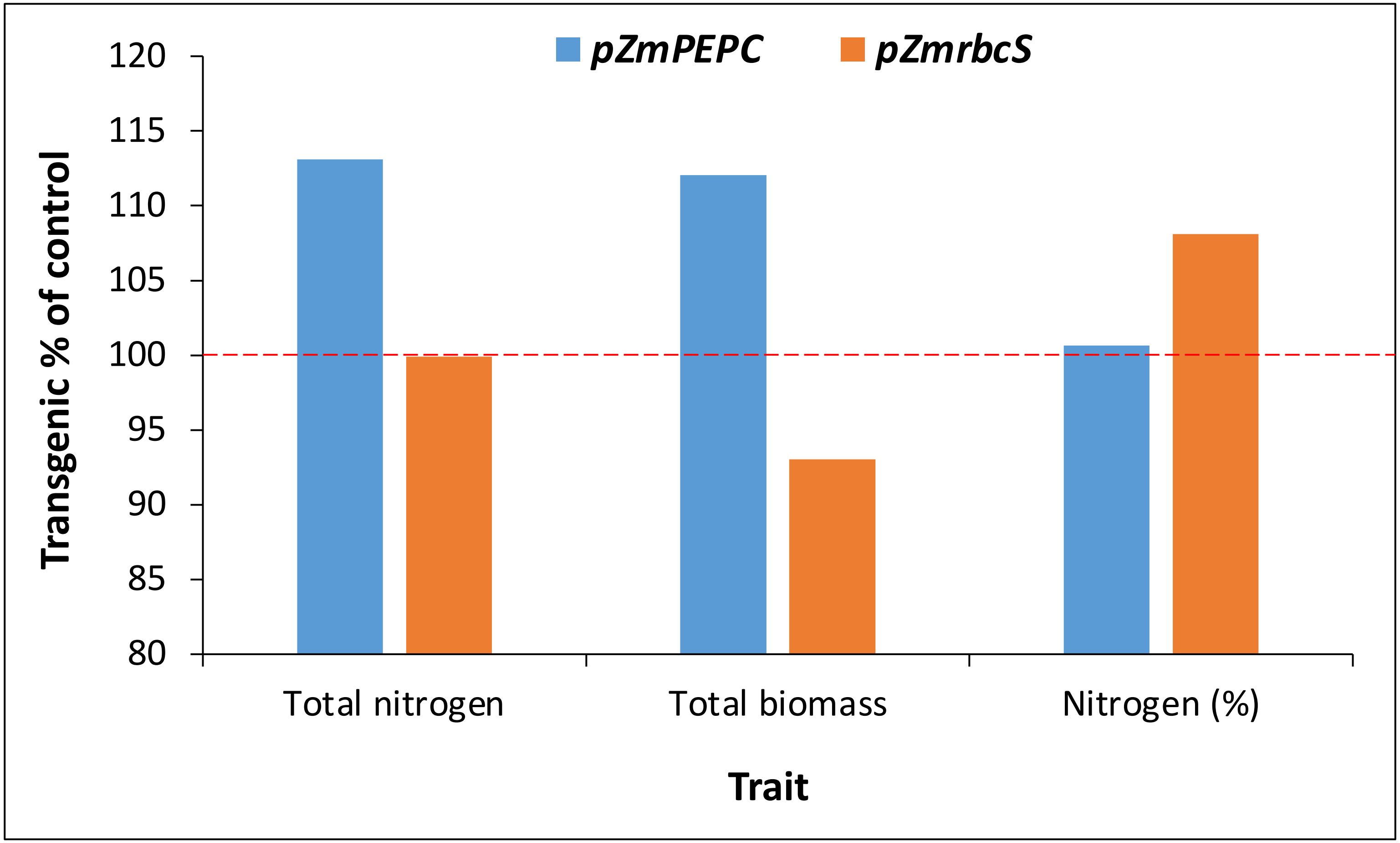

Table S1 Secondary traits for the transgenic maize events overexpressing ZmLOX6. Growing degree units (GDU) to silking (SLK) and pollen shed (SHD), plant height (PLHT in inches), and grain moisture content (MST in %) at maturity are shown. The transgenic expression of ZmLOX6 did not significantly affect any of these plant characteristics under optimal growing conditions or drought stress imposed at flowering.

Trait	Event	JH2008	WO2008FS	WO2009OPT	WO2009FS
GDUSLK	CN	1451	1490	1300	1270
	24	1450	1490	1300	1270
	25		1490	1300	1260
	28	1451		1300	1260
	32	1450	1470	1300	1260
	34	1475		1300	1260
GDUSHD	CN	1451	1470	1320	1330
	24	1450	1470	1320	1310
	25		1510	1330	1360
	28	1450	1440	1320	1370
	32	1450	1490	1330	1350
	34	1475		1320	1360
PLHT	CN			159.5	132
	24			162	133
	25			159.5	133.5
	28			161	132.5
	32			159.5	132
	34			159	132.5
MST	CN	24.7	24.0	24.1	24.2
	24	24.9	24.3	22.0	22.7
	25	24.9	28.4	26.4	25.9
	28	24.7	23.8	22.8	24.5
	32	23.7	23.5	25.6	23.5
	34	25.2		24.1	23.6

Table S2 Seedling growth assay of transgenic events expressing *ZmLOX6* gene under the control of a bundle sheath-specific promoter, p*ZmrbcS* (a) or a mesophyll-specific promoter, p*ZmPEPC* (b).

a

***pZmrbcS*::*ZmLox6* event**	**Total nitrogen (mg)**	**Significance**	**Total mass (g)**	**Significance**	**Nitrogen (mg/g)**	**Significance**
Control	77.26		2.21		34.94	
51	77.55	NS	2.07	0.068	37.67	0.012*
53	74.67	NS	2.09	0.094	36.41	NS
54	86.70	0.015*	2.25	NS	38.84	<0.001*
55	68.15	0.018	1.84	<0.001	43.12	<0.001*
56	78.93	NS	2.03	0.026	38.90	<0.001*

b

***pZmPEPC*::*ZmLox6* event**	**Total nitrogen (mg)**	**Significance**	**Total mass (g)**	**Significance**	**Nitrogen (mg/g)**	**Significance**
Control 1	76.21		1.98		38.95	
22	79.75	NS	1.96	NS	41.22	0.062*
24	85.30	0.013*	2.32	<0.001*	37.27	NS
25	85.52	0.011*	2.16	0.025*	39.43	NS
28	79.64	NS	2.03	NS	39.45	NS
29	95.68	<0.001*	2.17	0.018*	44.08	<0.001*
Control 2	71.13		1.72		41.48	
30	84.19	<0.001*	2.05	<0.001*	42.04	NS
31	85.58	<0.001*	2.13	<0.001*	40.20	NS
32	80.06	0.004*	1.95	0.002*	41.11	NS
33	77.05	0.033*	2.01	<0.001*	38.26	0.004
34	80.46	0.002*	1.95	0.002*	41.71	NS
